# The effect of competition on the control of invading plant pathogens

**DOI:** 10.1111/1365-2664.13618

**Published:** 2020-04-17

**Authors:** Ryan T. Sharp, Michael W. Shaw, Frank van den Bosch

**Affiliations:** ^1^ Department of Sustainable Agriculture Sciences Rothamsted Research Harpenden Hertfordshire UK; ^2^ School of Agriculture, Policy and Development University of Reading Reading Berkshire UK; ^3^ Department of Environment & Agriculture Centre for Crop and Disease Management Curtin University Bentley, Perth WA Australia

**Keywords:** *Bemisia tabaci*, cassava mosaic disease, competitive release, disease control, dispersal, invasive species, trade, wave of advance

## Abstract

1. New invading pathogen strains must compete with endemic pathogen strains to emerge and spread. As disease control measures are often non‐specific, that is, they do not distinguish between strains, applying control not only affects the invading pathogen strain but the endemic as well. We hypothesize that the control of the invasive strain could be compromised due to the non‐specific nature of the control.
2. A spatially explicit model, describing the East African cassava mosaic virus‐Uganda strain (EACMV‐UG) outbreak, is used to evaluate methods of controlling both disease incidence and spread of invading pathogen strains in pathosystems with and without an endemic pathogen strain present.
3. We find that while many newly introduced or intensified control measures (such as resistant cultivars or roguing) decrease the expected incidence, they have the unintended consequence of increasing, or at least not reducing, the speed with which the invasive pathogen spreads geographically. We identify the controls that cause this effect and methods in which these controls may be applied to prevent it.
4. We found that the spatial spread of the invading strain is chiefly governed by the incidence at the wave front. Control can therefore be applied, or intensified, once the wave front has passed without increasing the pathogen's rate of spread.
5. When trade of planting material occurs, it is possible that the planting material is already infected. The only forms of control in this study that reduces the speed of geographic spread, regardless of the presence of an endemic strain, are those that reduce the amount of trade and the distance over which trade takes place.
6. *Synthesis and applications*. The best control strategy depends on the presence of competing endemic strains. Applying or intensifying the control can slow the rate of spread when absent but increase it if present. Imposing trade restrictions before the epidemic has reached a given area and intensifying other control methods only when the wave front has passed is the most effective way of both slowing down spread and controlling incidence when a competing endemic strain is present and is the safest approach when its presence is unknown.

## Graphical abstract

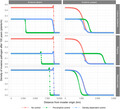

## 1 INTRODUCTION

Invasion of new pathogen strains is a major risk for both natural and agricultural systems. The conventional response when an emerging pathogen strain begins to cause significant losses to yield is to introduce new methods of control or intensify existing ones. While the reduction in impact on crop yield may be the primary focus from an individual grower's perspective, controlling the spatial spread of these invasive pathogen strains is of key importance to the entire grower community. Invasive pathogens may appear in a region by range expansion (Prospero, Grunwald, Winton, & Hansen, 2009; Stukenbrock, Banke, & McDonald, 2006), or new strains may emerge from pathogen strains endemic to the region; it is this mechanism in which resistance to controls develop (Hawkins, Bass, Dixon, & Neve, 2018). When an invasive strain emerges from an endemic, the invader must compete for resources with the endemic strain, as the endemic strain will already occupy part of the resource available (Abdullah et al., 2017; Read & Taylor, 2001). Control measures (e.g. pesticides, resistant cultivars or sanitation) generally do not distinguish between pathogen strains, therefore increasing control when a new strain emerges reduces the incidence of not only the invader, but of the endemic as well. Control therefore has the adverse effect of freeing up host resource that will benefit the invading strain, a process known as competitive release. The overall effect of control that affects both the endemic and the invader is unclear, however.

The spatial spread of invading species has long been studied. Originally studied by Fisher (1937) many studies have followed, further developing the mathematical theory behind the process (Thieme, 1977; van den Bosch, Metz, & Diekmann, 1990). Of these, there have also been studies considering a species invading into a competing resident species (Okubo, Maini, Williamson, & Murray, 1989; Skellam, 1951). Okubo et al. (1989) found that the spread of the invasive species, in this case the grey squirrel in the UK, was slower when spreading into an area with a competing resident species, the red squirrel. Tompkins, White, and Boots (2003) also studied this system but included disease from the parapoxvirus that was introduced with the grey squirrels. They found that the inclusion of the disease, which is particularly harmful to red squirrels, increased the rate of spread of the invader. An analogous study was performed by Moorcroft, Pacala, and Lewis (2006). Here the invasion of Beech into an area colonized by a close competitor, Hemlock, was studied. They found that infection by host‐specific pathogens lead to an increased rate of spread of the invader due to the invasive species escaping infection as it spread while the resident species was suppressed. In both these cases the disease was primarily acted upon the resident species, leading to competitive release and benefiting the invader. More recently in the medical literature, there have been studies of competitive release due to treatment of coinfected hosts, for example, the effect of treating malaria in hosts with both drug‐sensitive and drug‐resistant malaria strains allowing the resistant strain to increase (Hansen & Day, 2014; Wargo, Huijben, Roode, Shepherd, & Read, 2007). In all these cases, the control that is applied affects the resident species more than the invading one. It remains to be seen what the effect would be when an indiscriminate control, that affects both strains equally, is applied. The effect of competitive release in coinfecting plant pathogens and methods to reduce its impact on the spatial spread of invaders also appears to be unexplored.

In this paper, we explore this potential trade‐off of indiscriminate control on competing pathogen strains by modelling how the presence of competing endemic strains affects the spatial spread of an invading pathogen strain and investigate methods that can effectively control disease incidence as well as the invasive strain's speed of spread. We investigate this question in the context of the East African Cassava Mosaic Virus‐Uganda (EACMV‐UG) pandemic of cassava first identified as a distinct virus in Uganda in 1997 (Zhou et al., 1997).

One of the main diseases affecting African cassava is the cassava mosaic disease caused by the cassava mosaic virus (CMV). Surveys estimate average disease incidence across the continent to affect between 50% and 60% of fields with an average loss to yield of 24% (Legg, Owor, Sseruwagi, & Ndunguru, 2006) or 2.2 t/ha (given an average yield at the time of 7 t/ha; FAO, 2020). In East Africa there are six species of CMV (Fauquet & Stanley, 2003), the most prevalent of these are African cassava mosaic virus (ACMV) and East African cassava mosaic virus (EACMV; Legg & Fauquet, 2004). A new strain of EACMV, EACMV‐Uganda (EACMV‐UG), emerged in Uganda in the late 1980s (Otim‐Nape, Bua, & Baguma, 1994). This new strain was found to cause much more severe symptoms with yield losses estimated to be 1.7 t/ha in areas unaffected by the pandemic but 4.3 t/ha in pandemic areas (FAO, 2020; Legg et al., 2006). The strain has spread contiguously from Uganda and by 2005 covered over 2.5 million km^2^ across nine countries and has slowly replaced the endemic ACMV over time (Legg et al., 2006). Control of this disease therefore has the potential to greatly improve yields. A number of approaches have been used to control the disease. The main forms of control include roguing (the removal of infected plants), selection of disease‐free planting material and the use of resistant varieties (Kanju, Mtunda, Muhanna, Raya, & Mahungu, 2003). We examine how these control measures, among others, perform when applied to an invading strain that is in competition with strains endemic to the region.

## 2 MATERIALS AND METHODS

We developed a system of partial integro‐differential equations representing a cassava host, a whitefly vector and two strains of cassava mosaic virus: an endemic strain and an invading strain with a fitness advantage.

To track the spread of the invasive strain from the point of initial infection a one‐dimensional spatial component is included in the model. The virus can be spread in one of two ways, either by the whitefly vector, *Bemisia tabaci*, carrying the pathogen or through the planting of infected cuttings.

The full model is given as

(1) $$\begin{array}{cc} \frac{\partial H\left(x,\;t\right)}{\partial t} & \;=\;\varphi_{H}\left(x,\;t\right)\;-\;\omega H\left(x,\;t\right)\;-\;\lambda_{e}Z_{e}\left(x,\;t\right)H\left(x,\;t\right)\;-\;\lambda_{i}Z_{i}\left(x,\;t\right)H\left(x,\;t\right),\\ \frac{\partial I_{e}\left(x,\;t\right)}{\partial t} & \;=\;\varphi_{I_{e}}\left(x,\;t\right)\;+\;\lambda_{e}Z_{e}\left(x,\;t\right)H\left(x,\;t\right)\;-\;\left(\omega \;+\;\rho \right)I_{e}\left(x,\;t\right),\\ \frac{\partial I_{i}\left(x,\;t\right)}{\partial t} & \;=\;\varphi_{I_{i}}\left(x,\;t\right)\;+\;\lambda_{i}Z_{i}\left(x,\;t\right)H\left(x,\;t\right)\;-\;\left(\omega \;+\;\rho \right)I_{i}\left(x,\;t\right),\\ \frac{\partial Z_{e}\left(x,\;t\right)}{\partial t} & \;=\;-\alpha Z_{e}\left(x,\;t\right)\;+\;\gamma I_{e}\left(x,\;t\right)Y\left(x,\;t\right)\;+\;\delta_{e}\left(x,\;t\right),\\ \frac{\partial Z_{i}\left(x,\;t\right)}{\partial t} & \;=\;-\alpha Z_{i}\left(x,\;t\right)\;+\;\gamma I_{i}\left(x,\;t\right)Y\left(x,\;t\right)\;+\;\delta_{i}\left(x,\;t\right). \end{array}$$

It describes, at a given location, *x*, and time, *t*, the change in density of: the healthy host, *H*; host infected with the endemic strain, *I_e_*; host infected with the invading strain, *I_i_*; vector not carrying the virus, *Y*; vector carrying the endemic strain, *Z_e_*; and vector carrying the invading strain, *Z_i_*. Cassava is planted at a rate *φ_j_*(*x*, *t*) (where *j* = *H*, *I_e_* or *I_i_* for the healthy hosts and hosts infected with the endemic and invading virus strain, respectively) and harvested at a rate, *ω*. Infected crops can be controlled by roguing at a rate, *ρ*. A vector carrying the cassava mosaic virus, *Z_e_* or *Z_i_*, inoculates a host as it feeds on it at a rate given by *λ_e_* and *λ_i_* for the endemic and invading virus strain respectively. Likewise, a vector carrying no form of the virus, *Y*, acquires the virus at a rate given by *γ*, as it feeds on an infected host, *I_e_* or *I_i_*. Vector death rate is given by *α*. Vector dispersal is given by *δ_j_*(*x*, *t*) (where *j* = *e* or *i* for the endemic and invading virus strain respectively).

### 2.1 Vector dynamics

We assume that the total vector population remains constant. With this assumption, the density (vectors/m) of non‐carrying vectors (*Y*) can be expressed as

(2) $$Y\left(x,\;t\right)\;=\;P\left(b,\;\alpha ,\;K\right)\;-\;Z_{e}\left(x,\;t\right)\;-\;Z_{i}\left(x,\;t\right),$$

where *P* is the equilibrium density of the vector population, and *Z_e_* and *Z_i_* are the densities of the vector carrying the endemic and invading strains, respectively. To calculate *P* the whitefly dynamics of Holt, Jeger, Thresh, and Otim‐Nape (1997) are incorporated, that is, vector birth rate is assumed to be density‐dependent and death rate is assumed to be density‐independent. The whitefly dynamics that lead to the equilibrium vector density are therefore given by

(3) $$\frac{\partial \tilde{P}\left(x,\;t\right)}{\partial t}\;=\;b\tilde{P}\left(1\;-\;\frac{\tilde{P}}{K}\right)\;-\;\alpha \tilde{P},$$

where $\tilde{P}$ is the total vector density state variable; *b* is vector birth rate; *α* is vector death rate; and *K* is the density above which no vector reproduction takes place. Equilibrium vector density is therefore given by

(4) $$P\left(b,\;\alpha ,\;K\right)\;=\;\left\{\begin{array}{cc} \frac{\left(b\;-\;\alpha \right)K}{b} & \text{if}\;b\;\geq \;\alpha ,\\ 0 & \text{otherwise}. \end{array}\right.$$

### 2.2 Vector dispersal

Vector dispersal is expressed as the following

(5) $$\begin{array}{cc} \delta_{e}\left(x,\;t\right) & \;=\;m\left[\int f_{e}\left(x\;-\;y\right)Z_{e}\left(y,\;t\right)dy\;-\;Z_{e}\left(x,\;t\right)\right],\\ \delta_{i}\left(x,\;t\right) & \;=\;m\left[\int f_{i}\left(x\;-\;y\right)Z_{i}\left(y,\;t\right)dy\;-\;Z_{i}\left(x,\;t\right)\right], \end{array}$$

for the endemic and invading strains respectively. The dispersal rate, *m*, describes the frequency with which the vector disperses. The integral determines the number of vectors immigrating to location *x* from the densities of the vector at all other locations *y* and the distance between points *x* and *y* according to a dispersal kernel *f_e_* or *f_i_*. The final term, *mZ*, determines the number of vectors emigrating from point *x*. We used the Laplace distribution as a dispersal kernel as it has been found to be a good model of biological dispersion for many different organisms, including dispersing insects (Lewis & Pacala, 2000). The distribution gives rise to waves that advance at a constant speed, as observed for EACMV‐UG (Otim‐Nape & Thresh, 2006), and is sufficiently fat‐tailed to match published studies on the migration and dispersal of *B. tabaci* (Byrne, Rathman, Orum, & Palumbo, 1996; Fauquet & Fargette, 1990; Isaacs & Byrne, 1998). The dispersal kernel takes the following form

(6) $$f\left(x\;-\;y|D\right)\;=\;\frac{1}{D\sqrt{2}}\exp\left(-\frac{\left|x\;-\;y\right|\sqrt{2}}{D}\right),$$

where *D* is the standard deviation of the distribution.

### 2.3 Cassava planting

Cassava is planted continuously at a constant total rate given by *σ*, vegetatively propagated from cuttings of prior harvests. We assume that there are always sufficient cuttings available and that they are not limited due to, for example, a poor harvest. As it is the root that is consumed and not the stem, it is reasonable to assume that there is an oversupply of stems. Cassava of the three states of infection is planted at rates *φ_j_*(*x*, *t*) which are determined by

(7) $$\begin{array}{cc} \varphi_{H}\left(x,\;t\right) & \;=\;\sigma \left\{\theta \;+\;\left(1\;-\;\theta \right)\left[\left(1\;-\;\zeta \right)\frac{H\left(x,\;t\right)}{\eta \left(x,\;t\right)}\;+\;\zeta \frac{\int f_{\zeta }\left(x\;-\;y\right)H\left(y,\;t\right)dy}{\int f_{\zeta }\left(x\;-\;y\right)\eta \left(y,\;t\right)dy}\right]\right\},\\ \varphi_{I_{e}}\left(x,\;t\right) & \;=\;\sigma \left(1\;-\;\theta \right)\left[\left(1\;-\;\zeta \right)\frac{\left(1\;-\;p\right)I_{e}\left(x,\;t\right)}{\eta \left(x,\;t\right)}\;+\;\zeta \frac{\int f_{\zeta }\left(x\;-\;y\right)\left(1\;-\;p\right)I_{e}\left(y,\;t\right)dy}{\int f_{\zeta }\left(x\;-\;y\right)\eta \left(y,\;t\right)dy}\right],\\ \varphi_{I_{i}}\left(x,\;t\right) & \;=\;\sigma \left(1\;-\;\theta \right)\left[\left(1\;-\;\zeta \right)\frac{\left(1\;-\;p\right)I_{i}\left(x,\;t\right)}{\eta \left(x,\;t\right)}\;+\;\zeta \frac{\int f_{\zeta }\left(x\;-\;y\right)\left(1\;-\;p\right)I_{i}\left(y,\;t\right)dy}{\int f_{\zeta }\left(x\;-\;y\right)\eta \left(y,\;t\right)dy}\right]. \end{array}$$

Cuttings will be in one of three states: healthy (*H*) or infected with either the endemic (*I_e_*) or invading strain (*I_i_*). These cuttings come from one of three sources (locally from a previous crop, trade with a neighbour or a ‘clean seed system’). A percentage of total cuttings may come from a grower's previous crop at location *x* (with probability, 1 − *ζ*) or growers may trade cuttings with one another (with probability, *ζ*). Trade is modelled in the same way that vector dispersal is modelled, with an integral to define the movement of cuttings to a location *x* from the host densities at all other locations *y* and the distances between the locations *x* and *y* according to a dispersal kernel *f_ζ_* with a trade‐specific standard deviation, *D_ζ_*. These two sources, however, have the potential to vertically transmit the disease through the planting of infected cuttings. Growers can pre‐treat cuttings or select the healthy cuttings to prevent this; the probability that infected cuttings are removed prior to planting is modelled by the parameter, *p*. The total density of cuttings that are available to be sourced for the next generation is therefore given by $\begin{array}{c} \eta \left(x,\;t\right)\;=\;H\left(x,\;t\right)\;+\;\left(1\;-\;p\right)\left(I_{e}\left(x,\;t\right)\;+\;I_{i}\left(x,\;t\right)\right) \end{array}$ and the proportions of cuttings that are either healthy, infected with the endemic strain or infected with the invading strain that are sourced from the previous crop at location *x* are then given by $\begin{array}{c} H\left(x,t\right)/\eta \left(x,t\right) \end{array}$ , $\begin{array}{c} \left(1\;-\;p\right)I_{e}\left(x,\;t\right)/\eta \left(x,\;t\right) \end{array}$ and $\begin{array}{c} \left(1\;-\;p\right)I_{i}\left(x,\;t\right)/\eta \left(x,\;t\right) \end{array}$ respectively. Thirdly, cuttings may be sourced through a ‘clean seed system’. This is a system designed to prevent the planting of infected cuttings by providing farmers with clean cuttings that have been sourced from fields with extremely rigorous disease control (Legg, 2011). The percentage of cuttings sourced from a clean seed system is given by the parameter, *θ*.

### 2.4 Control

The effects of seven types of control were tested: cultivar resistance, reduced planting, roguing, removal of infected cuttings, using a clean seed system, limiting trade (either by restricting the amount of cuttings traded or the distance that they are traded) of infected cuttings and insecticides. These control measures are modelled by adjusting model parameters from the default parameter set appropriately. Cultivar resistance can be modelled by reducing either the inoculation rate, *λ* or the acquisition rate, *γ*. A reduction in planting rate, *σ*, can model the effect of reducing planting densities as it directly influences the total host density in the model. Limiting trade of infected cuttings can be achieved in two ways: either by reducing the amount of cuttings sourced through trade (*ζ*), or by reducing the distance that cuttings are traded (*D_ζ_*). Both forms of control are explored in this paper. Control in the form of roguing, the removal of infected cuttings before planting, the proportion of cuttings obtained through a clean seed system and the use of insecticides is modelled by increasing *ρ*, *p*, *θ* and *α* respectively.

### 2.5 Analysis

Analytical methods were used to calculate the (equilibrium) densities of disease incidence and the remaining healthy host both before and after invasion, as well as the speed with which the invasive strain spreads through its environment. The analytical method used to calculate the invasion speed is given in Appendix S1 in Supporting Information.

Numerical simulations were also performed to implement density‐dependent control measures in which control is initiated once the density (or incidence) of plants infected by the invasive strain exceeds a certain threshold. The model (Equation 1) was calculated using an adaptive time step method and the dispersal terms were calculated using convolution over a discretized spatial domain. The initial conditions of the state variables were set to their equilibrium values when the invader is absent. The invasion is then initiated by setting $Z_{i}\left(x,\;t\;=\;0\right)\;=\;{\bar{Z}}_{e}$ where ${\bar{Z}}_{e}$ is the equilibrium value of *Z_e_* and $Z_{e}\left(x,\;t\;=\;0\right)\;=\;0$ when the endemic strain is present, and $Z_{i}\left(x,\;t\;=\;0\right)\;=\;\bar{Y}/100$ when endemic strain is absent as this is roughly equivalent to ${\bar{Z}}_{e}$ .

Table 1 summarizes the parameters used in the model along with the default values used. A full discussion of how these were derived can be found in Appendix S2. Note that all the default values are greater than zero. Therefore, in the following sections in which we explore the effect of each control, there is some fixed background level of control occurring from other sources.

**TABLE 1:** Table summarizing parameters of the model and the default values tested. See Appendix S2 for how these values were chosen

Symbol	Description	Value
Crop parameters		
*ω*	Harvest rate (day^−1^)	0.003
*σ*	Planting rate (plants m^−1^ day^−1^)	0.003
*ρ*	Rate of removal (sanitation, roguing) of infected plants (day^−1^)	0.003
*λ_e_*, *λ_i_*	Inoculation rate of the endemic and invading pathogen strains to the host due to a carrying vector, respectively (m vector^−1^ day^−1^)	0.0064, 0.008
Cutting selection		
*θ*	Percentage of cuttings from clean seed system (CSS)	0.05
*ζ*	Percentage of cuttings (not from clean seed system) traded	0.5
*p*	Percentage of infected cuttings removed before planting	0.4
Vector parameters		
*b*	Vector birth rate (day^−1^)	0.2
*α*	Vector death rate (day^−1^)	0.12
*P*	Equilibrium vector density (vectors/m)	50
*γ*	Acquisition rate of pathogen by non‐carrying vectors (m plant^−1^ day^−1^)	0.004
Dispersal parameters		
*m*	Vector dispersal rate (day^−1^)	0.025
*D*	Vector dispersal kernel standard deviation (m)	1,000
*D_ζ_*	Trade dispersal kernel standard deviation (m)	30,000

Of the seven forms of control tested, we present here the results of the effect of cultivar resistance, roguing and trade (see Appendix S3 for reduced planting, removal of infected cuttings, using a clean seed system and insecticides). Cultivar resistance and roguing are two of the main forms of control that are used in East Africa (Legg et al., 2006), and we also present the effect of reducing trade as it behaved differently to the other forms of control.

In addition to the results presented here, a number of additional cases were examined and are presented in Supporting Information. In Appendix S4, numerical simulations of time‐dependent control were performed in which control was applied periodically in an attempt to capitalize on an initial dip observed in invasion speeds immediately following the intensification of control. In Appendix S5, the effect of roguing with replacement was explored, in which a new cutting was immediately planted following the removal of an infected plant. In Appendix S6, the model was modified to better incorporate the dynamics of another key disease of cassava, cassava brown streak virus (CBSV). In Appendix S7, the disease transmission terms were modified to include the effects of frequency‐dependent disease transmission. In Appendix S8, we explored a case where the invader's increased fitness increases its detectability, and therefore has a higher roguing rate. Finally, in Appendix S9 we alter the fitness advantage of the invasive strain.

## 3 RESULTS

### 3.1 Analytical results

#### 3.1.1 Cultivar resistance

**FIGURE 1:**
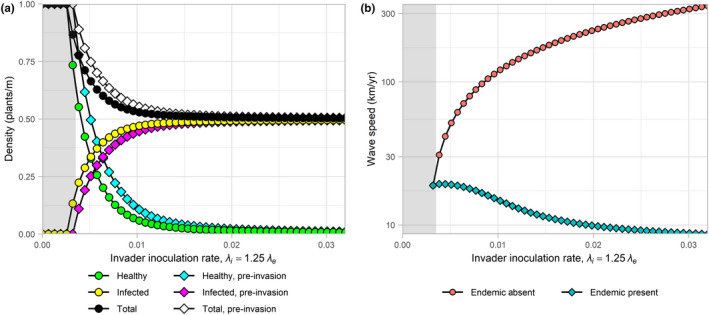
One‐way sensitivity analysis investigating the effect of planting‐resistant cultivars by decreasing the inoculation rate of the endemic strain, *λ_e_*, and the fitter invading strain, *λ_i_*, on: (a) healthy, infected and total host densities both pre‐ and post‐invasion; and, (b) speed of spread (log scale) of the invading pathogen strain when invading a region with either the endemic strain present or absent. Grey regions indicate areas in which the endemic strain has been removed entirely from the system prior to invasion due to the extreme levels of control being applied. In this situation the two invasion speeds are equivalent

The effect of changes in the inoculation rate is shown in Figure 1, the effect of changes in the acquisition rate can be found in Appendix S3, the results of which were analogous to that observed in Figure 1.

Introducing resistant cultivars reduces the final incidence regardless of whether the endemic strain is present or not and increases both healthy and total host. The effect on total host is due to the reduced levels of roguing that occurs due to a reduction in infected plants. Post‐invasion, the model predicts that the invading strain will completely replace the endemic strain; the final densities when either the endemic strain is present or absent are therefore equal. When no endemic strain is present the speed of spread of the invasive strain is reduced with increased control. When the endemic strain is present, however, the speed of invasion is increased with increased control. This shows that the effect of reduced competition can outweigh the reduced rate of increase due to control and result in a net benefit to the invasive strain. Invasion speed increased from 8.6 km/year, at the lowest level of cultivar resistance tested, up to 19.4 km/year as the level of resistance was increased. With further control the endemic strain can no longer persist and is removed from the system (indicated by the grey region). At this point, the speed of spread of the invading strain when the endemic strain is present is equal to that in the case when it is absent. Increasing control even further eventually results in the invading strain also being removed from the system.

#### 3.1.2 Roguing

**FIGURE 2:**
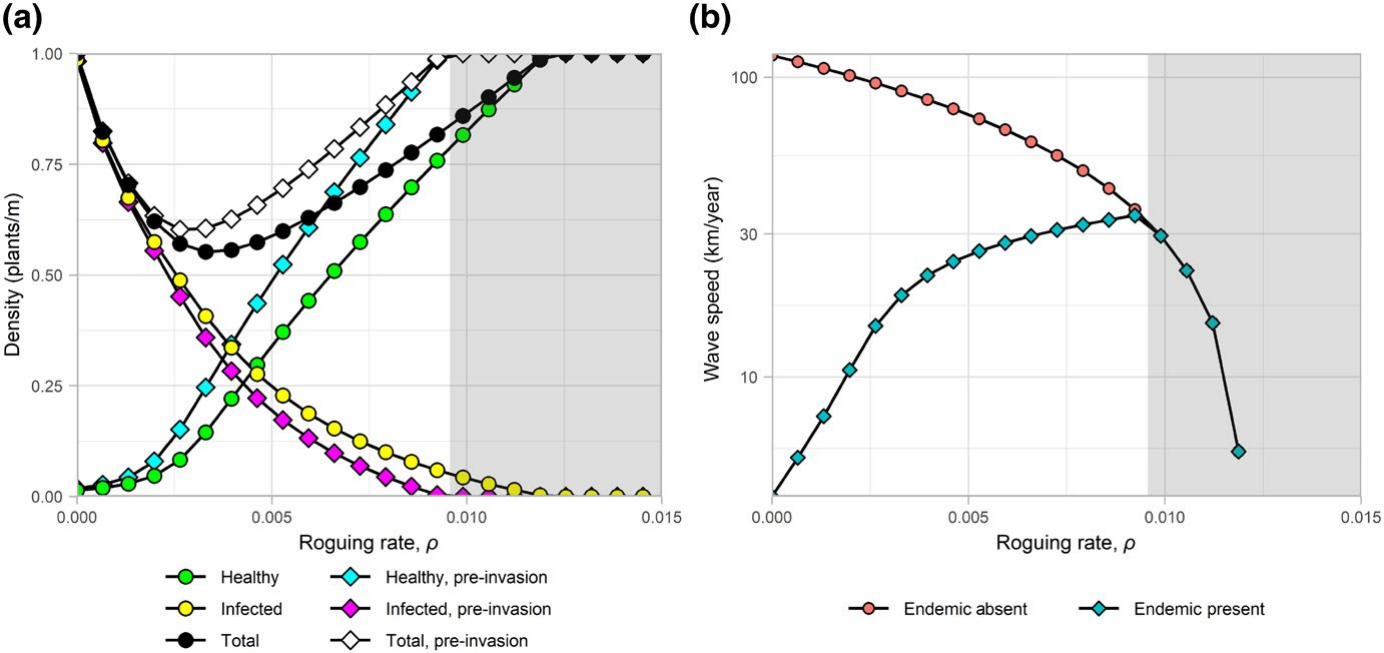
One‐way sensitivity analysis investigating the effect of increasing the rate in which infected plants are removed from a crop, *ρ*, on: (a) healthy, infected and total host densities both pre‐ and post‐invasion; and (b) speed of spread (log scale) of the invading pathogen strain when invading a region with the endemic strain present and absent. Grey regions indicate areas in which the endemic strain is removed entirely from the system due to the extreme levels of control being applied. In this situation the two invasion speeds are equivalent

The effects of making changes to the roguing rate, *ρ*, are similar to those observed when changing cultivar resistance (Figure 2). Again, there is a decrease in the infected density and an increase in the healthy density with increasing control. A pronounced effect on the invasion speeds as the roguing rate is increased is observed, with large decreases to invasion speed when the endemic strain is absent but large increases to invasion speed when the endemic strain is present. We therefore again observe a net benefit to the invasive strain due to reduced competition outweighing the reduced rate of increase due to control.

#### 3.1.3 Trade

**FIGURE 3:**
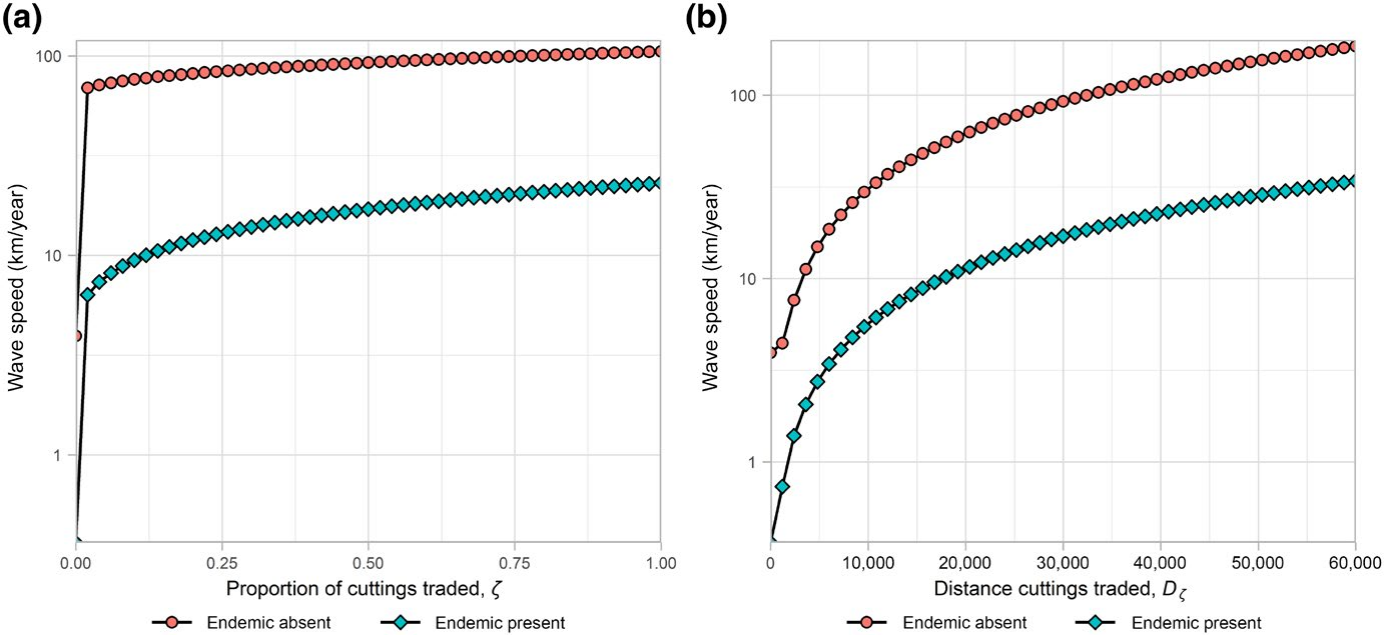
One‐way sensitivity analysis investigating the effect of: (a) reducing the proportion of cuttings traded, *ζ*; and, (b) reducing the distance cuttings which are traded, *D_ζ_*, on the speed of spread (log scale) of the invading pathogen strain when invading a region with the endemic strain present and absent

Limiting trade is a potential way of controlling disease as it reduces the trading and spread of infected cuttings. This form of ‘control’ can be implemented by either reducing the proportion of cuttings sourced or by reducing the distance that cuttings are traded (Figure 3). The invasion speed decreases as trade decreases both when the endemic strain is present and when it is absent, as this directly limits the spread of cuttings infected with the invasive pathogen strain.

However, because the densities from the analytical calculations are calculated when the system is spatially homogenous (i.e. before the introduction of the new strain and when the new strain has finished invading), trade‐based control measures have no effect.

#### 3.1.4 Analytical results summary

The effect of control measures on the final density and the rate of invasion is summarized in Table 2. When cultivar resistance or roguing is applied or intensified, the speed of spread of invasion increases with it when there is an endemic strain present. This behaviour is also observed with the additional control measures tested in Appendix S3. The only form of control where this does not occur are those measures that reduce trade. The effect on final disease incidence is more straightforward however; the effect of control is unaffected by the presence of an endemic strain.

**TABLE 2:** Summary of the effect increasing control measures has on both final disease incidence and rate of invasion

Control method	Endemic strain absent		Endemic strain present	
	Final disease incidence	Rate of invasion	Final disease incidence	Rate of invasion
Cultivar resistance	↓	↓	↓	↑
Roguing	↓	↓	↓	↑
Reduced trade	—	↓	—	↓

The differences in the wave speed's magnitude observed between the cases where the endemic strain is present and where it is absent demonstrates how the presence of an endemic strain, and the competitive effect that it creates, greatly reduces the speed with which the invading pathogen can invade.

### 3.2 Density‐dependent control

The analytical results show what happens when control is applied everywhere, regardless of whether the disease is present at a given location or not. As, in practice, control will likely be applied or increased with increases in incidence, the effect of applying control in a density‐dependent manner was tested. Applying control when the density of the invader exceeds a certain threshold—which can be determined by regular surveying and diagnosis (Sseruwagi, Sserubombwe, Legg, Ndunguru, & Thresh, 2004)—exercises control at the centre of the wave and not at the advancing wave front where the pathogen is present but rare. If speed can be slowed in such a manner, then the cost of control could be reduced as less area would be under increased control.

To illustrate the effect of density‐dependent control on invasion speeds, numerical simulations were performed where control is applied or intensified at a given location once the levels of the invasive strain reached a certain threshold (*I_i_* = 5 × 10^–2^). We compare these results to the case where control is not applied or intensified (‘No Control’) and to the case modelled by the analytical results in which control is even applied or intensified ahead of the advancing wave front (‘pre‐emptive control’).

**FIGURE 4:**
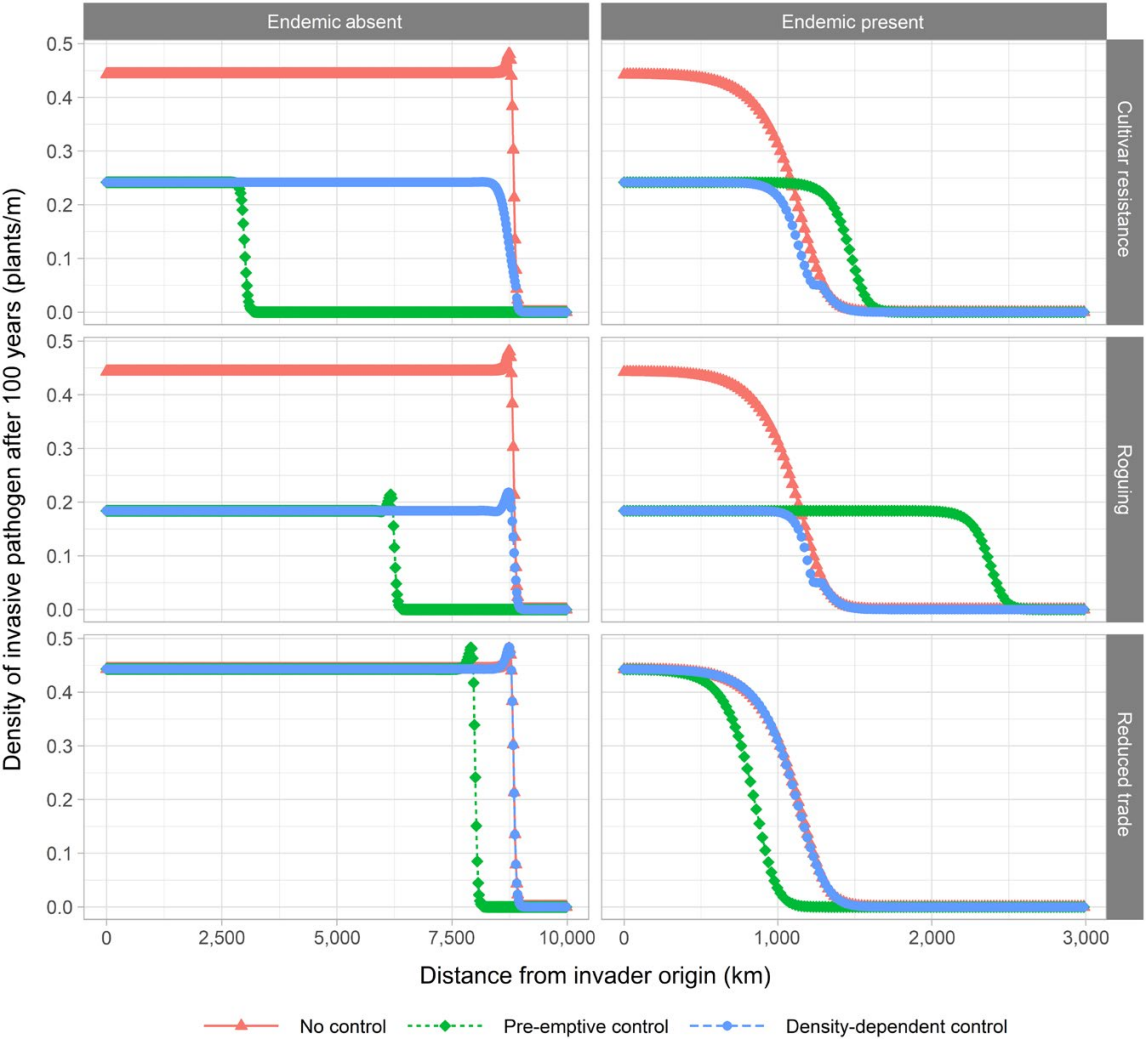
Numerical simulation results of the progress of the invasive strain 100 years after its introduction

Figure 4 plots the progress of the invasive strain 100 years from its initial introduction. When there is no endemic strain present we see that the results are again qualitatively similar, regardless of the form of control applied. We see that while pre‐emptive control slows the speed of spread, density‐dependent control has no effect on it. When the endemic strain is present, we observe that the speed of invasion is increased when cultivar resistance or roguing is applied pre‐emptively and that the speed of invasion is decreased with reduced trade, as was the case with the analytical results. There is again no effect on invasion speed when any of the control measures are applied in a density‐dependent manner. The effect on density is the same regardless of whether the control was applied pre‐emptively or density‐dependently and regardless of the presence or absence of the endemic strain. This matches the analytical results.

## 4 DISCUSSION

We investigated the potential trade‐off between the reduced rate of increase of the invader and the reduced effect of competition due to control. The effect of increasing control measures against an invading pathogen strain on both disease incidence and rate of spread was analysed in both the presence and absence of endemic strains. The results presented in this paper consistently show that the presence of a competing endemic strain completely alters the effect of control on the spatial spread of the invasive strain.

In the modelled cases where the endemic strain was absent, the invader invades a system with no competitors. The pathogen therefore has the entire healthy population as a resource for growth. Applying control to this system restricts the ability of the pathogen population to grow, reducing disease incidence/severity and the speed of spread. When there is an endemic strain, there is initially a competing endemic pathogen strain which has reached its equilibrium density resulting in a relatively stable disease incidence. As an invasive strain enters the system, much of the healthy resource is already occupied by the endemic strain. As the invasive strain is fitter, it has a higher density at equilibrium than the endemic strain and can utilize more of the resource. Therefore, so long as there is some healthy host remaining prior to invasion, there is still some resource for it to use to invade. Lack of available host when the endemic strain is present severely reduces the rate of infection of the host, however. It is because of this that such a large difference can be seen between the invasion speeds when the endemic is present or when it is absent. This demonstrates the competitive pressure that the invader is under. When control of the endemic strain is intensified, the amount of healthy host available to the invader is increased. While this control still decreases the invader's density at equilibrium and restricts its rate of increase as when the endemic strain is absent, the additional healthy host generated due to the simultaneous control of the endemic strain offsets the reduction in the rate of growth.

Invasion speeds of the EACMV‐UG pandemic had been found to vary between 20 and 100 km/year (Legg et al., 2011). The cause of these very high invasion speeds has yet to be found. In light of the model results, competitive effects between pathogen strains may be playing a role in this discrepancy and studies investigating competition between pathogen strains could elucidate this issue.

Post‐invasion analytical results predict the final densities expected at a particular location, that is, when the invader has fully invaded an area and the system has reached equilibrium. In the model with the endemic strain, the invader completely replaces the endemic strain and, because the invasive strain was assumed to be fitter than the endemic, causes a larger incidence of diseased plants at equilibrium compared with the incidence prior to invasion. While EACMV‐UG has not been seen to completely replace ACMV yet, it is gradually replacing ACMV over time (Legg et al., 2006). For example, in central Uganda where the strain originated, in a period of 7 years the proportion of singly infected plants infected with EACMV‐UG was estimated to have increased from 33% in 1995 (Harrison, Zhou, Otim‐Nape, Liu, & Robinson, 1997) to 88% in 2002 (Sseruwagi, Rey, Brown, & Legg, 2004). While invasion speeds reacted in a counter‐intuitive manner to control, the effect of control on densities was straightforward: all the non‐trade‐based control measures increased the density of healthy hosts.

The effect of most of the control measures on densities and invasion speeds were similar, with the exception of measures that limited the trade of infected cuttings. These were the only control measures to reduce invasion speed when applied pre‐emptively with the endemic strain present; all other measures increased it. While applying the other forms of control pre‐emptively can slow down the speed of spread when the endemic strain is absent, they increase the speed of spread when the endemic strain is present. Therefore, if applying control pre‐emptively, care must be taken to ensure that there are no competitive effects at play.

As previously discussed, an issue with the analytical solutions is that control is applied even in regions that have yet to be affected by the invasive strain. Scenarios were therefore tested by simulation in which control was intensified after the invasive strain had invaded and reached a certain threshold. This is the more likely scenario in practice, as control is likely to be intensified once levels of the invasive strain are high enough to be detected. We found that the speed of spread of the invasive strain was no longer affected when control was applied in this way. Invasion speeds therefore appear to be critically dependent on the dynamics of the invasion wave's front. It is in this region where the effect of competition is strongest, and therefore where the application of control reduces competition the most. The effect on the final density was the same in both cases. This means that there are no negative side‐effects to density‐dependent control. As invasion speed appears to be critically dependent on the densities at the wave front, the best strategy for controlling both invasion speed and incidence when the presence of an endemic strain is unknown is to apply trade‐based control prior to invasion and then to relax these measures in favour of the others once the wave front has passed. When applying control in a density‐dependent manner, care must be taken when selecting an appropriate threshold. This form of control is an intermediate approach between pre‐emptive control (obtained by setting a threshold of zero) and not applying any control (obtained by setting the threshold equal to the equilibrium density). The effect of density‐dependent control can therefore vary.

As with any model, a number of assumptions were made. We investigated the effect of relaxing a number of these assumptions in Supporting Information, as detailed in Section 2.5. These results did not alter the conclusions drawn here and were therefore omitted. One case that we did not explore is the competitive or cooperative effects that can occur between strains as a result of coinfection (Abdullah et al., 2017). While a plant in our model may be infected with both pathogen strains, we do not track this, and we therefore assume that the strains continue to infect as they would in a singly infected plant. In the case of the EACMV‐UG epidemic, Colvin, Omongo, Maruthi, Otim‐Nape, and Thresh (2004) found a cooperative effect of control, as the strain infects much more readily in the presence of the endemic strain. Exploring this effect would require a new model that tracks the density of plants that are coinfected and we therefore did not test these additional effects in this study. We hypothesize though that the increase to the invader's speed of spread, due to increased control when the endemic strain is present, will be lessened or even removed entirely in pathogens that coinfect cooperatively and be greater in those that coinfect competitively.

There is increasing interest in the effect of competitive release (Hansen & Day, 2014; Moorcroft et al., 2006; Tompkins et al., 2003; Wargo et al., 2007). The controls applied to all of these studies, however, affect the endemic species more than the invasive one. In this study, our control measures were non‐specific, applying pressure to both invasive and endemic pathogen strains. Nevertheless, we still observed an increase in the rate of spread of the invasive strain.

To conclude, in this study a vector‐borne epidemic of a vegetatively propagated crop has been investigated. While the Ugandan cassava mosaic virus epidemic has been used as an example due to its current importance, the results are much more generally applicable as the model in its current form can apply to any vector‐borne pathogen. Further, while our model was formulated to describe the propagation of the disease through the planting of infected cuttings, it could also be used to model seed‐borne diseases. Limiting trade will of course only be effective on crops that can vertically transmit the disease, or in cases where the traded party is of the current generation (e.g. the movement of the whole plant). As it is competition between the pathogen populations that generates this trade‐off it is anticipated that the results, or similar ones, will apply in many situations where pathogens directly or indirectly (via host induced resistance) interact within a host population.

## AUTHORS' CONTRIBUTIONS

F.v.d.B. conceived the idea of this study; F.v.d.B. and R.T.S. developed the analytical framework of this work; R.T.S. carried out the simulations, analysed the data and led the writing of this manuscript; M.W.S. provided critical appraisal of model, results and draft manuscript. All authors contributed critically to the manuscript and gave final approval for publication.

## Supporting information

Appendix S1

Appendix S2

Appendix S3

Appendix S4

Appendix S5

Appendix S6

Appendix S7

Appendix S8

Appendix S9

## Data Availability

Data and code are available via Zenodo https://doi.org/10.5281/zenodo.3694205 (Sharp, Shaw, & van den Bosch, 2020).
