# Supplementary material for: The effect of competition on the control of invading plant pathogens
Source: J Appl Ecol. 2020 Apr 17;57(7):1403–12. doi: 10.1111/1365-2664.13618 (PMC7386929; doi:10.1111/1365-2664.13618)
Supplement: Supplementary file 1 — Appendix S1 [file JPE-57-1403-s001.pdf]

The effect of competition on the control of invading plant pathogens

Ryan T. Sharp[1,*], Michael W. Shaw[2] & Frank van den Bosch[3]

[1]*Department of Sustainable Agriculture Sciences, Rothamsted Research, Harpenden, Hertfordshire, AL5 2JQ, UK*

[2]*School of Agriculture, Policy and Development, University of Reading, Whiteknights, Reading, Berkshire, RG6 6AS, UK*

[3]*Department of Environment & Agriculture, Centre for Crop and Disease Management, Curtin University, Bentley 6102, Perth, Australia*

[*]**Author for correspondence - (ryan.sharp@rothamsted.ac.uk)**

Appendix S1. Analytical approximation

To calculate the invasion-speed, the system is approximated and linearised about the leading edge of the invasion. It is assumed that prior to invasion the system has reached equilibrium and is spatially homogenous ($H = \bar{H}$, $I_e = \bar{I}_e, Z_e = \bar{Z}_e, I_i = 0, Z_i = 0$). The density of the host and vector infected with the invading pathogen at the edge of the invasion wave is then given by $I_i = i_i$ and $Z_i = z_i$ such that $0 \leq |i_i|, |z_i| \ll 1$. The linearised system is then given by

$$\begin{aligned}\frac{\partial i_i(x,t)}{\partial t} &= \frac{\sigma(1-\theta)(1-p)}{\bar{H}+(1-p)\bar{I}_e}\left[(1-\zeta)i_i(x,t)+\zeta\int_{-\infty}^{\infty} f_\zeta(x-y)i_i(y,t)\,dy\right]+\lambda_i z_i(x,t)\bar{H} \\ &\quad -(\omega+\rho)i_i(x,t), \\ \frac{\partial z_i(x,t)}{\partial t} &= -\alpha z_i(x,t)+\gamma_i i_i(x,t)(P(\alpha,b,K)-\bar{Z}_e) \\ &\quad + m\left(\int_{-\infty}^{\infty} f_i(x-y)z_i(y,t)dy - z_i(x,t)\right).\end{aligned} \tag{1}$$

Substitution of an exponential decaying wave front into the above equation and looking for travelling wave solutions of the form

$$\begin{aligned} i_i(x-ct) &= C_{i_i}\exp\big(-a(x-ct)\big), \\ z_i(x-ct) &= C_{z_i}\exp\big(-a(x-ct)\big), \end{aligned} \tag{2}$$

where C_{i_i} and C_{z_i} represents the densities, a represents the slope of the advancing edge of the wave, and c is the wave speed, yields

$$\begin{aligned} caC_{i_i} &= \frac{\sigma(1-\theta)(1-p)}{\bar{H}+(1-p)\bar{I}_e}\left[(1-\zeta)C_{i_i} + \frac{\zeta \int_{-\infty}^{\infty} f_\zeta(x-y)C_{i_i}\exp\big(-a(y-ct)\big)\,dy}{\exp\big(-a(x-ct)\big)}\right] \\ &\quad + \lambda_i C_{z_i}\bar{H} - (\omega+\rho)C_{i_i}, \\ caC_{z_i} &= -\alpha C_{z_i} + \gamma_i C_{i_i}(P(\alpha,b,K) - \bar{Z}_e) \\ &\quad + m\left(\frac{\int_{-\infty}^{\infty} f_i(x-y)C_{z_i}\exp\big(-a(y-ct)\big)\,dy}{\exp\big(-a(x-ct)\big)} - C_{z_i}\right). \end{aligned} \tag{3}$$

This can be simplified by noting that

$$\begin{aligned} \frac{\int_{-\infty}^{\infty} f_\zeta(x-y)C_{i_i}\exp\big(-a(y-ct)\big)\,dy}{\exp\big(-a(x-ct)\big)} &= C_{i_i}\int_{-\infty}^{\infty} f_\zeta(x-y)\exp\big(a(x-y)\big)\,d(x-y) \\ &= C_{i_i}\hat{f}_\zeta(a), \\ \frac{\int_{-\infty}^{\infty} f_i(x-y)C_{z_i}\exp\big(-a(y-ct)\big)\,dy}{\exp\big(-a(x-ct)\big)} &= C_{z_i}\int_{-\infty}^{\infty} f_i(x-y)\exp\big(a(x-y)\big)\,d(x-y) \\ &= C_{z_i}\hat{f}_i(a), \end{aligned} \tag{4}$$

where $\hat{f}_\zeta(a)$, $\hat{f}_i(a)$ are the moment generating functions of the trade and vector dispersal kernels, respectively. As f_ζ and f_i are Laplace kernels with zero mean, the moment generating functions are given by

$$\hat{f}(a) = \frac{1}{1-\left(a\frac{D}{\sqrt{2}}\right)^2} \quad \text{for} \quad |a| < \frac{\sqrt{2}}{D}, \tag{5}$$

where *D* (i.e. either D_ζ or D_i) is the standard deviation of the Laplace kernel.

Equation 3 can therefore be represented in matrix form as

$$ca\begin{pmatrix} C_{i_i} \\ C_{z_i} \end{pmatrix} = A\begin{pmatrix} C_{i_i} \\ C_{z_i} \end{pmatrix}, \tag{6}$$

where

$$A = \begin{pmatrix} \frac{\sigma(1-\theta)(1-p)}{\bar{H}+(1-p)\bar{I}_e}\big[(1-\zeta)+\zeta\hat{f}_\zeta(a)\big] - (\omega+\rho) & \lambda_i\bar{H} \\ \gamma_i(P-\bar{Z}_e) & -\alpha + m\big[\hat{f}_i(a)-1\big] \end{pmatrix}. \tag{7}$$

The wave speed is then given by

$$c(a) = \frac{\xi_1(a)}{a}, \quad 8$$

where $\xi_1(a)$ is the leading eigenvalue of the coefficient matrix, A, which is calculated by solving the characteristic equation of equation 6, $L(a, c) = \det(A - \xi I) = 0$. The asymptotic wave speed of the epidemic is then given by

$$c^* = \min_{0<a<\min\frac{\sqrt{2}}{D_\zeta},\frac{\sqrt{2}}{D_i}} c(a), \quad 9$$

which can be obtained by solving

$$\frac{dc(a)}{da} = 0 : \; 0 < a < \min\frac{\sqrt{2}}{D_\zeta}, \frac{\sqrt{2}}{D_i} \quad 10$$

(van den Bosch, Zadoks & Metz, 1988; Neubert & Caswell, 2000; Bitsouni *et al.*, 2018).

References

Bitsouni, V., Trucu, D., Chaplain, M. A. J. & Eftimie, R. (2018). Aggregation and travelling wave dynamics in a two-population model of cancer cell growth and invasion. *Mathematical Medicine and Biology-a Journal of the Ima, 35*(4)**,** 541-577. 10.1093/imammb/dqx019.

Neubert, M. G. & Caswell, H. (2000). Demography and dispersal: Calculation and sensitivity analysis of invasion speed for structured populations. *Ecology, 81*(6)**,** 1613-1628. 10.1890/0012-9658(2000)081[1613:Dadcas]2.0.Co;2.

van den Bosch, F., Zadoks, J. C. & Metz, J. A. J. (1988). Focus expansion in plant disease. I: The constant rate of focus expansion. *Phytopathology, 78*(1)**,** 54-58.
