# Supplementary material for: The effect of competition on the control of invading plant pathogens
Source: J Appl Ecol. 2020 Apr 17;57(7):1403–12. doi: 10.1111/1365-2664.13618 (PMC7386929; doi:10.1111/1365-2664.13618)
Supplement: Supplementary file 2 — Appendix S2 [file JPE-57-1403-s002.pdf]

The effect of competition on the control of invading plant pathogens

Ryan T. Sharp[1, *], Michael W. Shaw[2] & Frank van den Bosch[3]

[1]*Department of Sustainable Agriculture Sciences, Rothamsted Research, Harpenden, Hertfordshire, AL5 2JQ, UK*

[2]*School of Agriculture, Policy and Development, University of Reading, Whiteknights, Reading, Berkshire, RG6 6AS, UK*

[3]*Department of Environment & Agriculture, Centre for Crop and Disease Management, Curtin University, Bentley 6102, Perth, Australia*

[*]**Author for correspondence - (ryan.sharp@rothamsted.ac.uk)**

Appendix S2. Parameter selection

Cassava is usually harvested 10–22 months after planting (Silvestre, 1989) but can be harvested as early as 6 months after planting. Assuming a constant rate of harvesting, the harvesting rate, ω, was taken to be between 0.002–0.004 day^{-1} (corresponding to a harvest approximately 8–17 months after planting) with a default value of 0.003 day^{-1}. The model has a maximum plant density of 1 plant m^{-1} when planting rate is equal to harvesting rate, which is the typical planting density of cassava. The same values used to define ω were therefore used to define the planting rate, σ.

A range of 0–0.033 day^{-1} was used for the roguing rate, ρ, which corresponds respectively to a crop never being rogued to one being entirely rogued of infected plants once every 2 months. A default value of 0.003 day^{-1} is assumed corresponding to a crop being rogued roughly once every 6 months.

The proportion of cuttings taken from a clean seed system was chosen to be low, $\theta = 0.05$, as clean seed systems are still in development and are not yet widely adopted (Legg, 2011). A study on the farming practices in three districts of Uganda revealed that on average approximately 50% of cuttings were sourced through trade (Otim-Nape, Bua & Baguma, 1994). We therefore estimated that of the remaining cuttings not sourced through a clean seed system, 50% are obtained through trade, i.e. $\zeta = 0.5$. The same study also showed that on average just under 40% of growers selected for healthy cuttings prior to planting. We used this to estimate the default value of p, the percentage of infected cuttings removed prior to planting.

Using the data collated by Fishpool and Burban (1994), Holt *et al.* (1997) estimated the birth rate of *B. tabaci* to be within the range of 0.1–0.3 day^{-1} with a default of 0.2 day^{-1} and the death rate, due to both biotic and abiotic factors, to be within the range 0.06–0.18 day^{-1} with a default value of 0.12 day^{-1}. Similar estimates are used here, but the range of the death rate tested is modified to incorporate the spraying of insecticides. Death rates larger than 0.18 day^{-1} are therefore tested, and the upper limit of the range is chosen to match the upper limit of the birth rate, as when the death rate is equal to the birth rate the equilibrium total vector density governed by equation 3 of the manuscript is zero. Larger death rates are therefore irrelevant.

Legg (1995) estimated the mean number of vectors per plant across various sites in Uganda to be from 0 to 100 plant^{-1}. The parameter K was therefore set so that P is within this range.

Based on the work of Byrne (1999), a default standard deviation, D, of 1 km was chosen for the vector dispersal kernel. A vector dispersal rate, m, of 0.025 day^{-1} was estimated from Meng, Sabelis and Janssen (2012) by fitting the model

$$\frac{\partial \tilde{P}(x,t)}{\partial t} = r\tilde{P} + m\left(\int \frac{1}{D\sqrt{2}} \exp\left(-\frac{|x-y|\sqrt{2}}{D}\right) \tilde{P}(y,t)dy - \tilde{P}(x,t)\right) \qquad 1$$

to the data and estimating m using the above estimate for D and estimating r from population data in Meng, Sabelis and Janssen (2012).

The spread of EACMV-UG in Uganda was estimated to be 20–30 km yr^{-1} (Gibson, Legg & Otim-Nape, 1996; Otim-Nape *et al.*, 2000) with the advance around Lake Victoria estimated to be 24 km yr^{-1} around the eastern side and 38 km yr^{-1} around the western side (Legg, 2010). Setting the standard deviation of the dispersal kernel that governs distances that cuttings are traded to be 30km gave a rate of spread comparable to that observed.

Holt *et al.* (1997) estimated inoculation rates to be 0.008 vector^{-1} day^{-1} (with a range of 0.002–0.032 vector^{-1} day^{-1}) . Colvin *et al.* (2004) found that while EACMV-UG transmission was marginally lower than ACMV in singly infected plants (Maruthi *et al.*, 2002), in dually infected plants EACMV-UG transmission was much higher. To accommodate these differences, a conservative estimate is taken and the inoculation rate of the invader is set to be 1.25 times higher than the resident strain.

Fargette *et al.* (1990) studied ACMV in Ivory Coast and found that the proportion of *B. tabaci* that are infective is typically less than 2%. A further study by Colvin *et al.* (2004) investigating the EACMV-UG spread found the proportion infective to be less than 10%. An acquisition rate of 0.004 m plant^{-1} day^{-1} gave proportions of infective vectors in the model comparable to those observed.

References

Byrne, D. N. (1999). Migration and dispersal by the sweet potato whitefly, *Bemisia tabaci*. *Agricultural and Forest Meteorology, 97*(4), 309-316. 10.1016/s0168-1923(99)00074-x.

Colvin, J., Omongo, C. A., Maruthi, M. N., Otim-Nape, G. W. & Thresh, J. M. (2004). Dual begomovirus infections and high Bemisia tabaci populations: two factors driving the spread of a cassava mosaic disease pandemic. *Plant Pathology, 53*(5), 577-584. https://doi.org/10.1111/j.1365-3059.2004.01062.x.

Fargette, D., Fauquet, C., Grenier, E. & Thresh, J. M. (1990). The spread of African cassava mosaic-virus into and within cassava fields. *Journal of Phytopathology-Phytopathologische Zeitschrift, 130*(4), 289-302. 10.1111/j.1439-0434.1990.tb01179.x.

Fishpool, L. D. C. & Burban, C. (1994). *Bemisia tabaci*: the whitefly vector of African cassava mosaic geminivirus. *Tropical Science, 34*(1), 55-72.

Gibson, R. W., Legg, J. P. & Otim-Nape, G. W. (1996). Unusually severe symptoms are a characteristic of the current epidemic of mosaic virus disease of cassava in Uganda. *Annals of Applied Biology, 128*(3), 479-490. 10.1111/j.1744-7348.1996.tb07108.x.

Holt, J., Jeger, M. J., Thresh, J. M. & Otim-Nape, G. W. (1997). An epidemiological model incorporating vector population dynamics applied to African cassava mosaic virus disease. *Journal of Applied Ecology, 34*(3), 793-806. https://doi.org/10.2307/2404924.

Legg, J. P. (1995). The ecology of *Bemisia tabaci* (Gennadius)(Homoptera: Aleyrodidae), vector of African cassava mosaic geminivirus in Uganda. PhD Thesis, University of Reading.

Legg, J. P. (2010). Epidemiology of a whitefly-transmitted cassava mosaic geminivirus pandemic in Africa. In P.A. Stansly & S.E. Naranjo (Eds.) *Bemisia: Bionomics and Management of a Global Pest*, (pp. 233-257). Netherlands: Springer.

Legg, J. P. (2011). Developing clean seed systems for cassava in East and Central Africa. *IITA - R4D Review,* (6), 41-42.

Maruthi, M. N., Colvin, J., Seal, S., Gibson, G. & Cooper, J. (2002). Co-adaptation between cassava mosaic geminiviruses and their local vector populations. *Virus Research, 86*(1-2), 71-85. 10.1016/s0168-1702(02)00051-5.

Meng, R. X., Sabelis, M. W. & Janssen, A. (2012). Limited Predator-Induced Dispersal in Whiteflies. *PLoS ONE, 7*(9). 10.1371/journal.pone.0045487.

Otim-Nape, G. W., Bua, A. & Baguma, Y. (1994). Accelerating the transfer of improved production technologies: controlling African cassava mosaic virus disease epidemics in Uganda. *African Crop Science Journal, 2*(4), 479-495.

Otim-Nape, G. W., Bua, A., Thresh, J. M., Baguma, Y., Ogwal, S., Ssemakula, G. N., Acola, G. & Byabakama, B. (2000). *The current pandemic of cassava mosaic virus disease in East Africa and its control*. Chatham, UK: NARO/NRI/DFID publication.

Silvestre, P. (1989). *Cassava*. London, UK: Macmillan Education.
