# Supplementary material for: The effect of competition on the control of invading plant pathogens
Source: J Appl Ecol. 2020 Apr 17;57(7):1403–12. doi: 10.1111/1365-2664.13618 (PMC7386929; doi:10.1111/1365-2664.13618)
Supplement: Supplementary file 3 — Appendix S3 [file JPE-57-1403-s003.pdf]

The effect of competition on the control of invading plant pathogens

Ryan T. Sharp[1, *], Michael W. Shaw[2] & Frank van den Bosch[3]

[1]*Department of Sustainable Agriculture Sciences, Rothamsted Research, Harpenden, Hertfordshire, AL5 2JQ, UK*

[2]*School of Agriculture, Policy and Development, University of Reading, Whiteknights, Reading, Berkshire, RG6 6AS, UK*

[3]*Department of Environment & Agriculture, Centre for Crop and Disease Management, Curtin University, Bentley 6102, Perth, Australia*

[*]**Author for correspondence - (ryan.sharp@rothamsted.ac.uk)**

Appendix S3. Effects of other forms of control

Cultivar resistance: acquisition rate

The inoculation rate and acquisition rate produce largely similar results as host densities, invasion speeds and the overall dynamics are similar. However, acquisition rates have a large effect on the proportion of infectious vectors whereas inoculation rate has very little effect. Large inoculation rates mean that a relatively small vector density can produce the same amount of infection as a larger density of less infectious vectors.

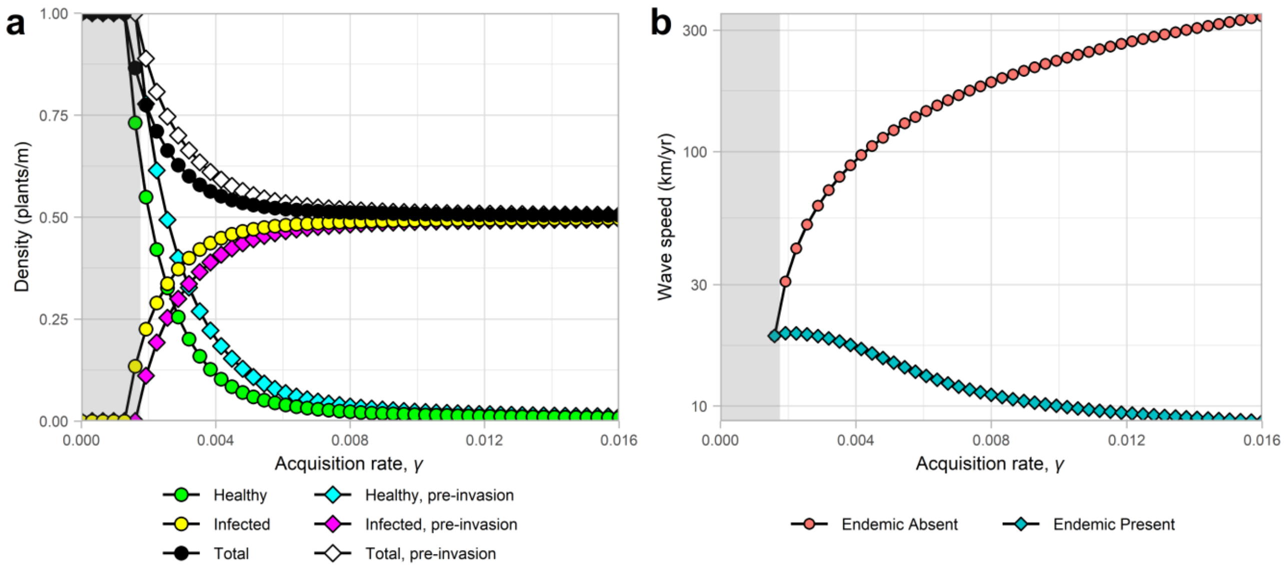

Figure 1 – One-way sensitivity analysis investigating the effect of planting resistant cultivars by decreasing the acquisition rate, γ, on: (a) healthy, infected and total host densities both pre- and post-invasion; and, (b) speed of spread (log scale) of the invading pathogen strain when invading a region with either the endemic strain present or absent. Grey regions indicate areas in which the endemic strain is removed entirely from the system due to the extreme levels of control being applied. In this situation the two invasion speeds are equivalent.

Reduced planting

The effect of reducing the amount of crop planted is shown in figure 2. Reducing the planting rate reduces total density as expected but also reduces the proportion of the crop that is infected. This results in a net growth to the healthy host. The invading strain again completely replaces the endemic strain. Invasion speed dynamics are similar to that seen when planting resistant cultivars: speed decreases with control in the single-strain model but increases with control in multi-strain model. Finally, for high levels of control, the endemic is first wiped out, and the invasion speed of the multi-strain model is equivalent to the single-strain model. As control is increased further the invader is eventually removed from the system.

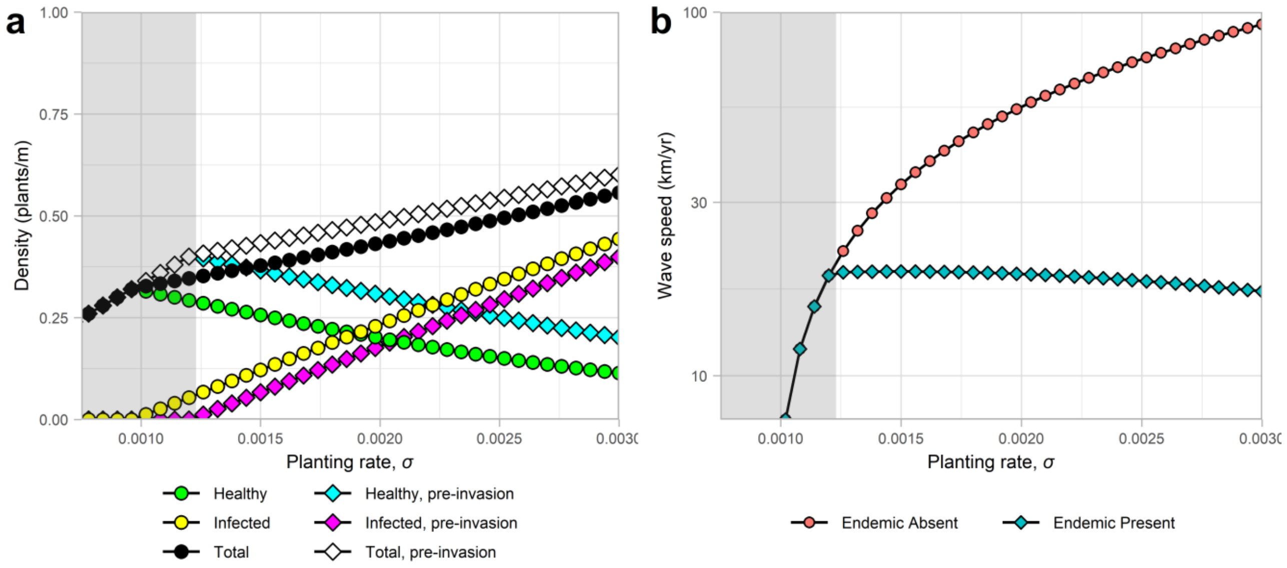

Figure 2 – One-way sensitivity analysis investigating the effect of reducing planting density by decreasing the planting rate, σ, on: (a) healthy, infected and total host densities both pre- and post-invasion; and, (b) speed of spread (log scale) of the invading pathogen strain when invading a region with either the endemic strain present or absent. Grey regions indicate areas in which the endemic strain is removed entirely from the system due to the extreme levels of control being applied. In this situation the two invasion speeds are equivalent.

Insecticide

The spraying of insecticides controls vector populations by increasing the vector death rate. The effect of changes to the vector death rate is shown in figure 3.

Increasing the vector death rate decreased final incidence and increased healthy and total densities as expected. It was also observed that when the endemic strain is removed prior to invasion, increasing control decreases the speed of the invader's spread. When the endemic is present, an increase in invasion speed is again observed.

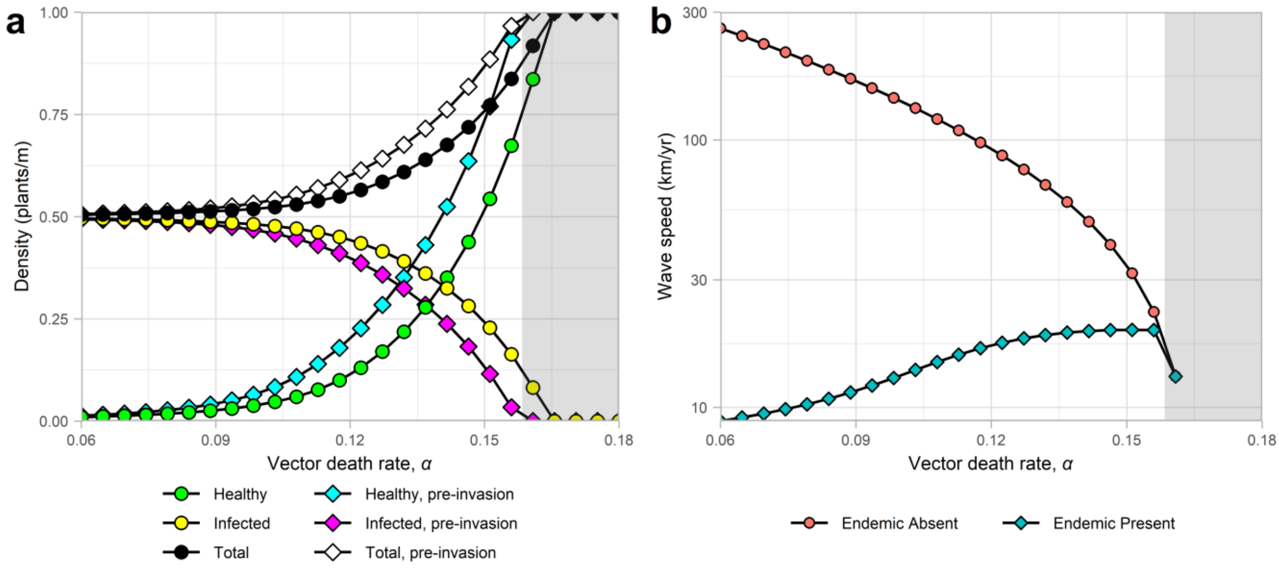

Figure 3 – One-way sensitivity analysis investigating the effect of spraying insecticides by increasing the vector death rate, α, on: (a) healthy, infected and total host densities both pre- and post-invasion; and, (b) speed of spread (log scale) of the invading pathogen strain when invading a region with either the endemic strain present or absent. Grey regions indicate areas in which the endemic strain is removed entirely from the system due to the extreme levels of control being applied. In this situation the two invasion speeds are equivalent.

Clean seed systems

The effect of changing the cuttings sourced through a clean seed system were tested, and the results shown in figure 4.

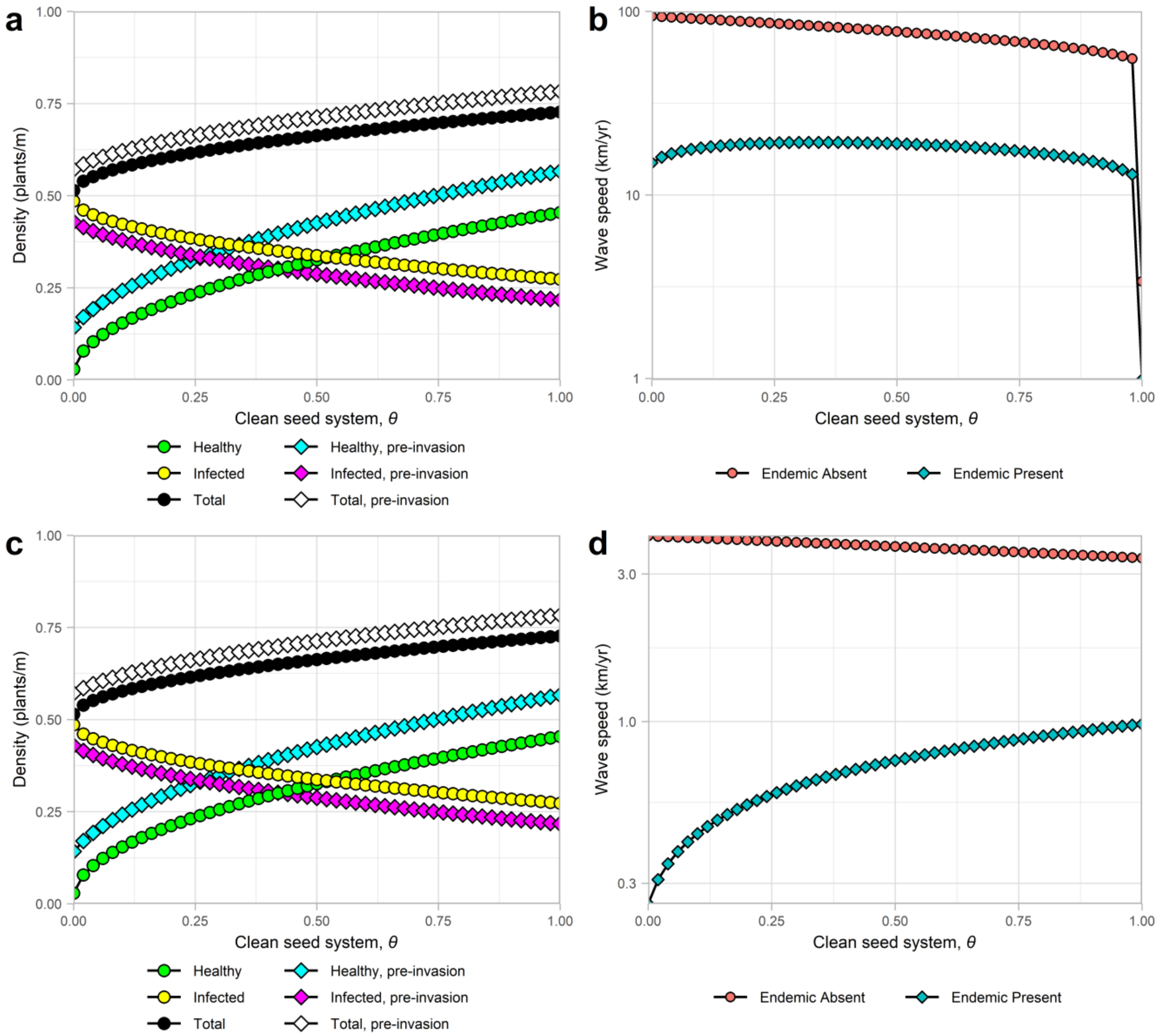

Figure 4 – One-way sensitivity analysis investigating the effect of increasing the proportion of cuttings sourced through a clean seed system, θ, on: healthy, infected and total host densities both pre- and post-invasion (figures (a) & (c)); and, speed of spread (log scale) of the invading pathogen strain when invading a region with either the endemic strain present or absent (figures (b) & (d)). Figures (a) & (b) uses the default parameter set and figures (c) & (d) plots dynamics when none of the cuttings are sourced through trade ($\zeta = 0$).

Sourcing more cuttings through a clean seed system increased the density of healthy hosts. Single-strain invasion speed decreased as expected. Multi-strain invasion speed initially increased for low levels of control but decreased as the proportion of cuttings sourced through a clean seed system was increased (figure 4(b)). This is because as control is increased, the system approaches a scenario in which cuttings are sourced entirely through a clean seed system ($\theta = 1$). This reduces the proportion of cuttings that are sourced from

the grower's own crop and, more importantly, the proportion received through trade. It is this reduction in trade that reduces the invasion speed. This can be seen by stopping trade altogether (figure 4(c) & (d)). Though at a greatly reduced level, a monotonic increase to invasion speed is now seen as the cuttings sourced through a clean seed system is increased. ζ is perhaps more aptly described as the proportion of non-CSS (clean seed system) cuttings traded. The total cuttings sourced therefore either come from the clean seed system, given by θ, through trade of a neighbours own cuttings, given by $\zeta(1-\theta)$, or sourced locally from one's own cuttings, given by $(1-\zeta)(1-\theta)$. By changing θ in the one-way sensitivity analyses the number of cuttings traded were also inadvertently varied. Keeping actual cuttings traded fixed and increasing θ therefore removes the effect of trade on invasion speed and incidence entirely and allows the effect of employing a clean seed system alone to be observed (albeit by reducing the proportion of cuttings sourced locally).

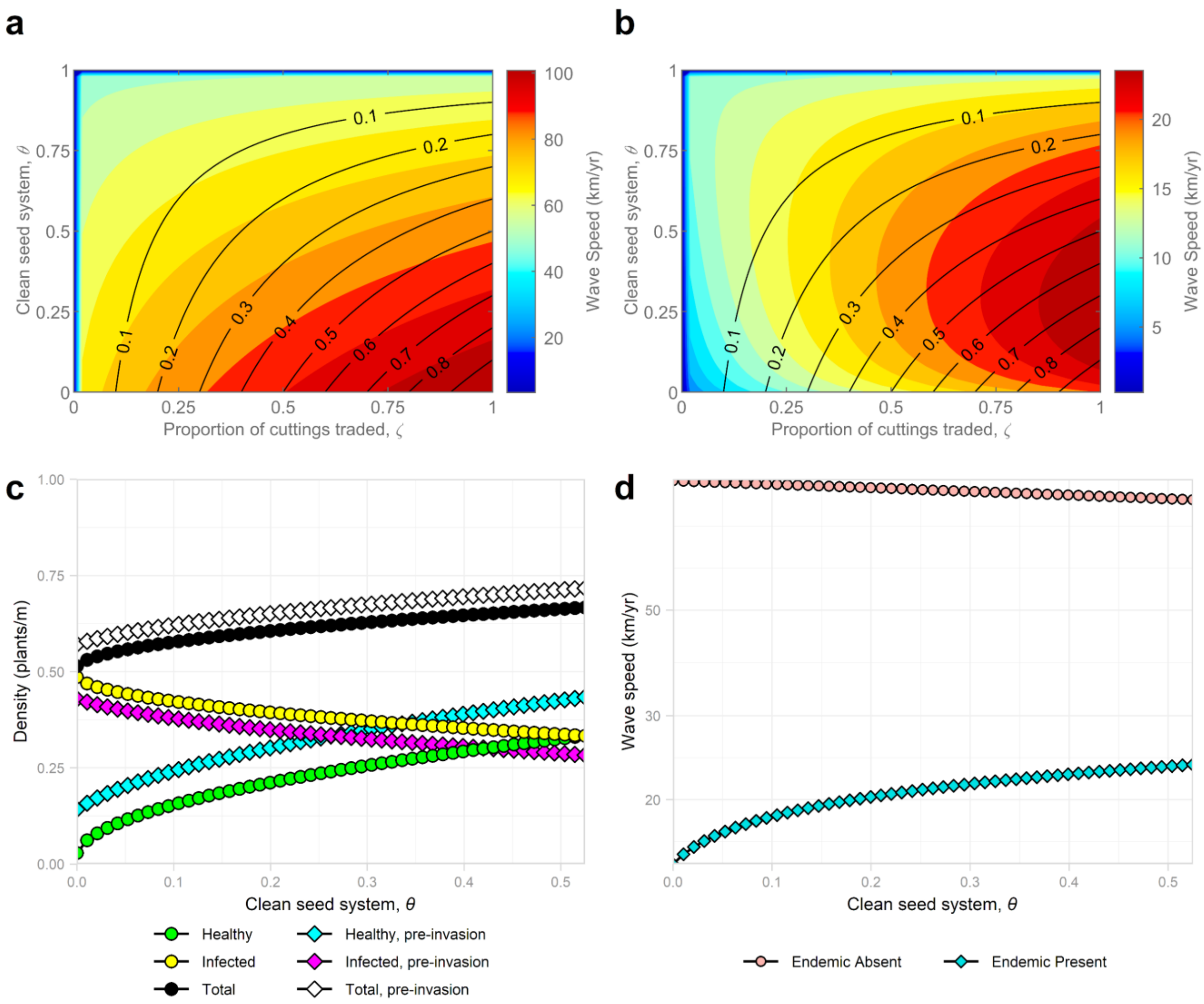

Figure 5 - Figures (a) & (b) plot two-way sensitivity analyses investigating the effect on invasion speed in the single-strain model (figure (a)) and the multi-strain model (figure (b)) by changing the proportion of cuttings sourced through either trade or a clean seed system. Black contours indicate the actual proportion of cuttings sourced through trade. Figures (c) & (d) plots a one-way sensitivity analysis investigating the effect of increasing the proportion of cuttings sourced through a clean seed system, θ, while actual cuttings traded are kept fixed at the default value ($0.475 = \zeta(1-\theta)$) on: (c) healthy, infected and total host densities both pre- and post-invasion; and, (d) speed of spread (log scale) of the invading pathogen strain when invading a region with either the endemic strain present or absent.

Figure 5(a) & (b) plot for the single- and multi-strain models respectively, two-way sensitivity analyses of the effect of changes to the proportion of clean 'seed' cuttings, θ, and the proportion of traded cuttings, ζ. The black contour lines give the actual cuttings traded, $\zeta(1-\theta)$. By fixing this value and following the black contour up the y-axis the effect of increasing cuttings sourced through a clean seed system on the invasion speed can be observed for any proportion of cuttings traded. By doing this, it can be seen that increasing the proportion of clean cuttings increases invasion speed monotonically for any proportion of cuttings traded. To further illustrate this point, figure 5(c) & (d) plots a one-way sensitivity analysis testing the effect of

changes to the proportion of clean cuttings, where traded cuttings are fixed at the default value of 0.475, on densities and invasion speeds respectively.

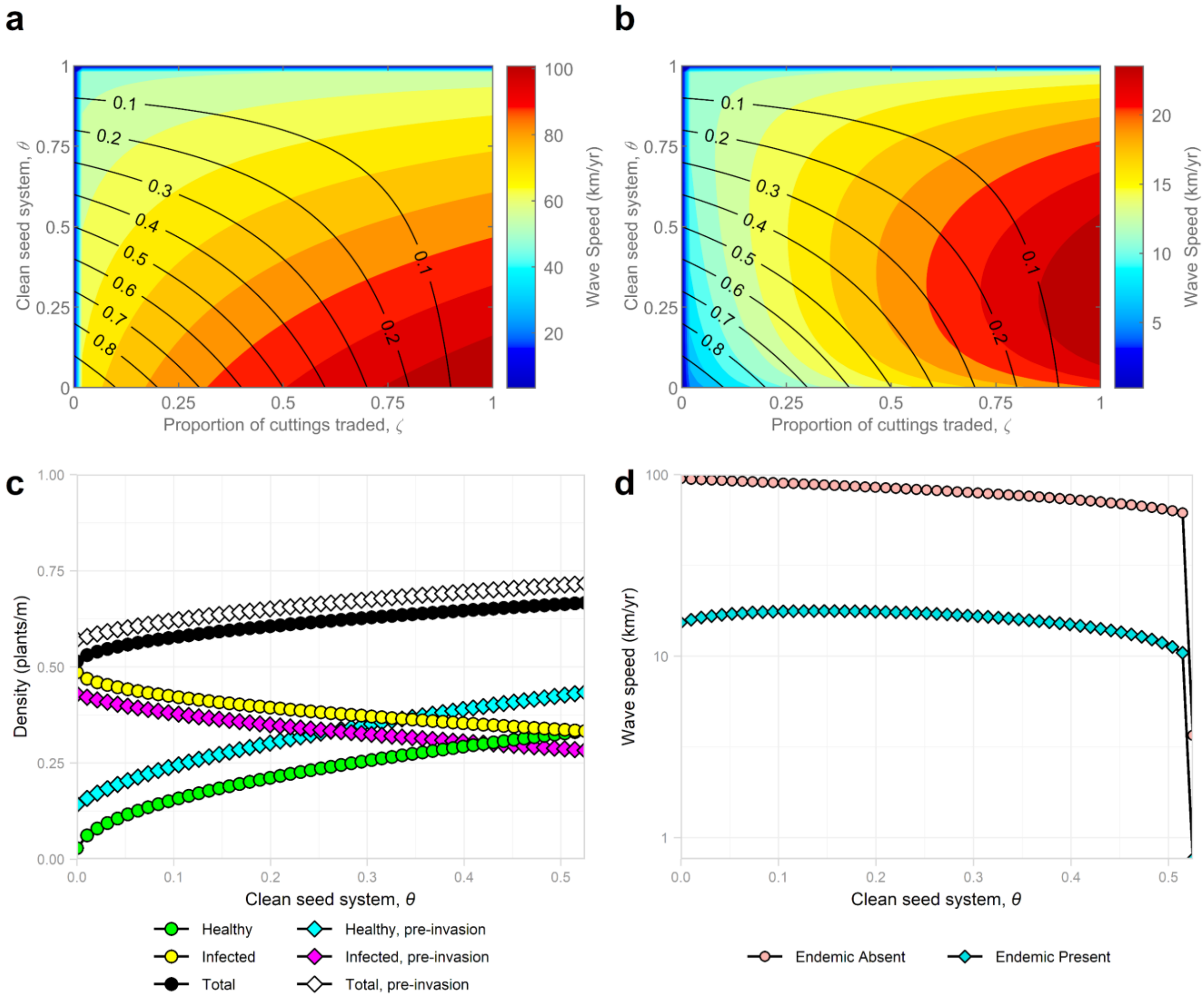

Figure 6 – Figures (a) & (b) plot two-way sensitivity analyses investigating the effect on invasion speed in the single-strain model (figure (a)) and the multi-strain model (figure (b)) by changing the proportion of cuttings sourced through either trade or a clean seed system. Black contours indicate the proportion of cuttings sourced locally from the previous crop. Figures (c) & (d) plots a one-way sensitivity analysis investigating the effect of increasing the proportion of cuttings sourced through a clean seed system, θ, while locally sourced cuttings are kept fixed at the default value ($0.475 = (1-\zeta)(1-\theta)$) on: (c) healthy, infected and total host densities both pre- and post-invasion; and, (d) speed of spread (log scale) of the invading pathogen strain when invading a region with either the endemic strain present or absent.

An issue with applying changes to the proportion of cuttings traded, ζ, in this model formulation is that a decrease in ζ corresponds to an increase in the proportion of cuttings used locally from a grower's own crop. This is a suitable characteristic of the model in some cases; however, it is likely that a grower may forego trade in favour of purchasing cuttings from a clean 'seed' system. A two-way sensitivity analysis is given in figure 6(a) & (b) in which changes are made to ζ and the proportion of total cuttings obtained through a clean

‘seed’ system, θ. The black contour lines correspond to proportions of cuttings taken locally (i.e. from the previous crop; given by $(1-\theta)(1-\zeta)$). By following the contour lines up the y-axis the effect of replacing traded cuttings for clean seed, while keeping proportions of local cuttings fixed, on invasion speed and incidence can be observed. Figure 6(c) & (d) plots an example trajectory through the two-way sensitivity analyses whereby the proportion of local cuttings is fixed at 0.475. In doing this a return to the original dynamics is observed, in which multi-strain invasion speed increases for low θ and decreases for high θ.

Removal of infected cuttings

Figure 7 plots the effect of changes to the proportion of infected cuttings removed before planting, p. Dynamics are similar to that seen when changes are made to the proportion of clean cuttings. While trade still occurs when all infected cuttings are removed prior to planting, the spread of any infected cuttings through trade is stopped. Figure 7(c) & (d) plots the sensitivity analysis when there is no trade, where a monotonic increase in the multi-strain invasion speed is again observed.

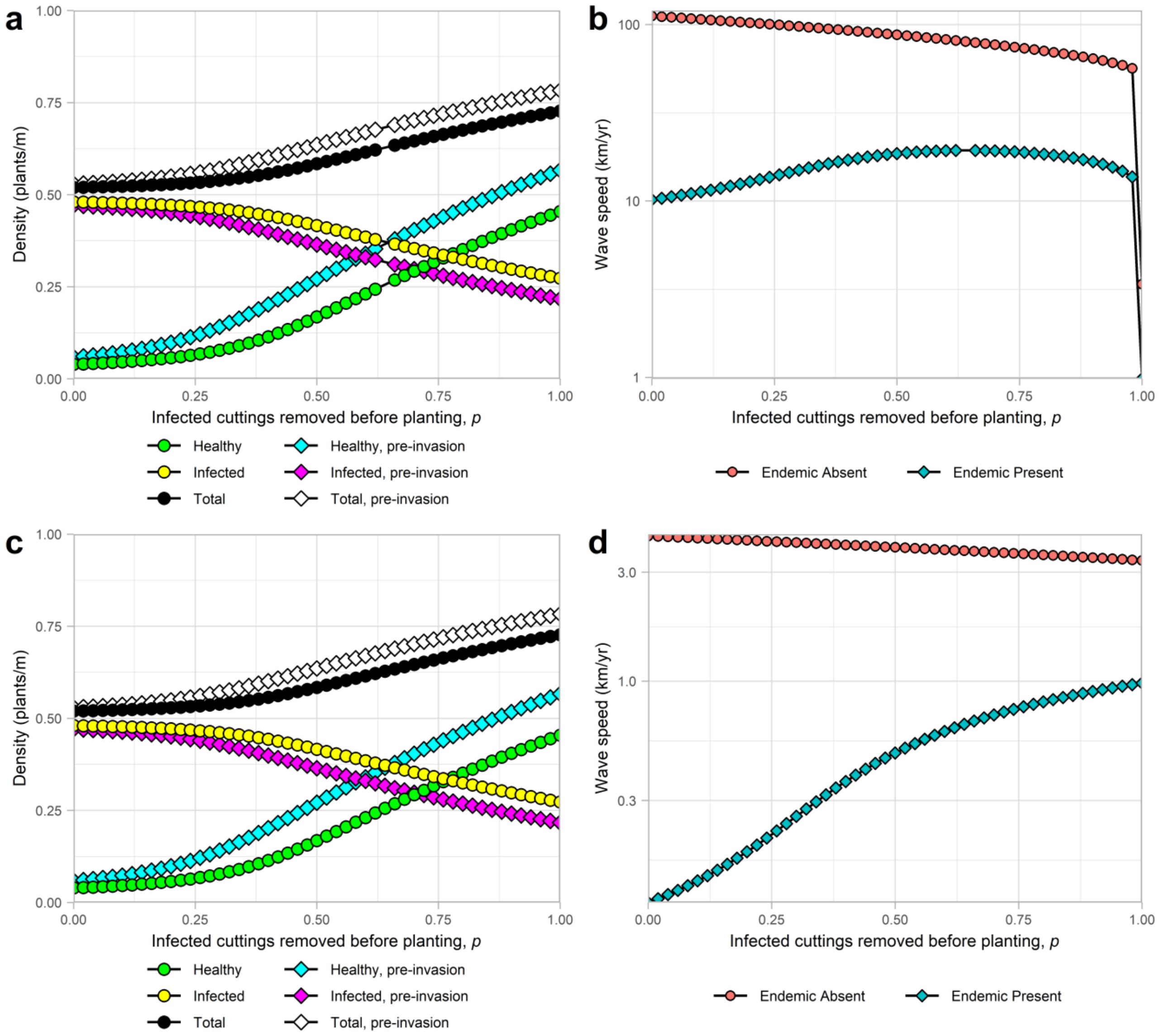

Figure 7 – One-way sensitivity analysis investigating the effect of increasing the proportion of infected cuttings that are removed before planting, p, on: healthy, infected and total host densities both pre- and post-invasion (figures (a) & (c)); and, speed of spread (log scale) of the invading pathogen strain when invading a region with either the endemic strain present or absent (figures (b) & (d)). Figures (a) & (b) uses the default parameter set and figures (c) & (d) plots dynamics when none of the cuttings are sourced through trade $(\zeta = 0)$.
