# Supplementary material for: The effect of competition on the control of invading plant pathogens
Source: J Appl Ecol. 2020 Apr 17;57(7):1403–12. doi: 10.1111/1365-2664.13618 (PMC7386929; doi:10.1111/1365-2664.13618)
Supplement: Supplementary file 4 — Appendix S4 [file JPE-57-1403-s004.pdf]

The effect of competition on the control of invading plant pathogens

Ryan T. Sharp[1, *], Michael W. Shaw[2] & Frank van den Bosch[3]

[1]*Department of Sustainable Agriculture Sciences, Rothamsted Research, Harpenden, Hertfordshire, AL5 2JQ, UK*

[2]*School of Agriculture, Policy and Development, University of Reading, Whiteknights, Reading, Berkshire, RG6 6AS, UK*

[3]*Department of Environment & Agriculture, Centre for Crop and Disease Management, Curtin University, Bentley 6102, Perth, Australia*

[*]**Author for correspondence - (ryan.sharp@rothamsted.ac.uk)**

Appendix S4. Time-dependent control

There was generally a drop in invasion speeds observed in the two-strain simulations when control measures such as cultivar resistance or roguing are first stepped up, even though these treatments eventually increase invasion speed. One might assume that the use of these control measures would still be effective if control were applied periodically in order to generate this initial reduction in speed. To test this, figure 1 plots the results from applying control periodically, where control is applied for one year and relaxed the next. Relaxing roguing, for example, in the two-strain model causes a jump in invasion speed in the other direction. The transient decreases and increases do not necessarily cancel: the effect, in the case shown in figure 1, is a net increase to invasion speed. While there may be cases in which a net decrease is possible, the benefit of such controls is small in comparison to density-dependent control.

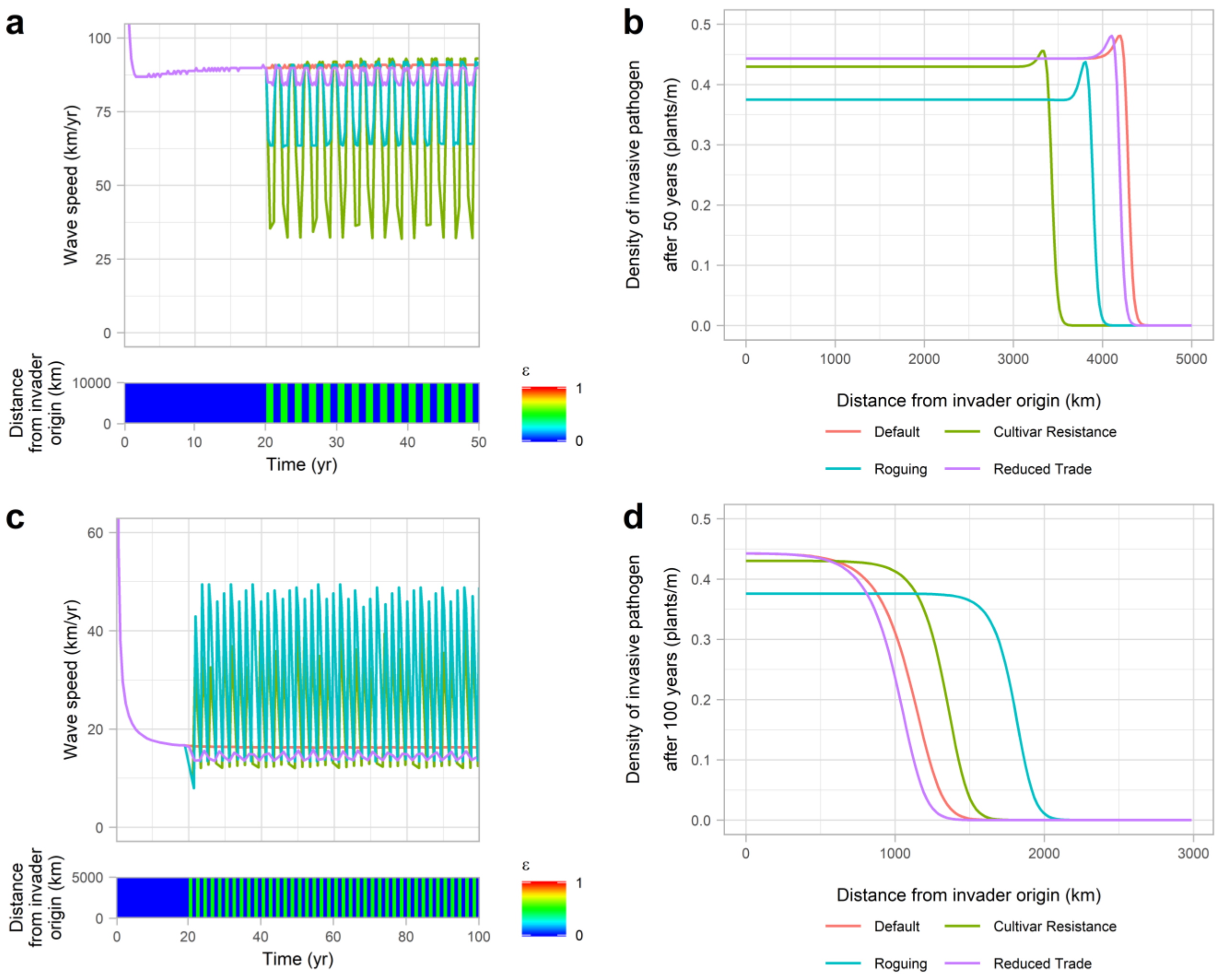

Figure 1 – Simulation results comparing the effects of increasing the proportions of resistant cultivars used (green), increasing roguing (blue) and reducing proportions of cuttings traded (purple) for a period of one year and then relaxing control for one year on: invasion speeds over time (figures (a) & (c)); and, the final density of hosts infected with the invasive pathogen strain over space, calculated 50 (figure (b)) and 100 years (figure (d)) after the invader first arrived for the single-strain and multi-strain models, respectively. The bar beneath figure (c) indicates how control was applied in the temporal and spatial domains, where $\lambda_\varepsilon = (1-\varepsilon)\lambda$; $\rho_\varepsilon = \rho/(1-\varepsilon)$ and $\zeta_\varepsilon = (1-\varepsilon)\zeta$.
