# Supplementary material for: The effect of competition on the control of invading plant pathogens
Source: J Appl Ecol. 2020 Apr 17;57(7):1403–12. doi: 10.1111/1365-2664.13618 (PMC7386929; doi:10.1111/1365-2664.13618)
Supplement: Supplementary file 5 — Appendix S5 [file JPE-57-1403-s005.pdf]

The effect of competition on the control of invading plant pathogens

Ryan T. Sharp[1, *], Michael W. Shaw[2] & Frank van den Bosch[3]

[1]*Department of Sustainable Agriculture Sciences, Rothamsted Research, Harpenden, Hertfordshire, AL5 2JQ, UK*

[2]*School of Agriculture, Policy and Development, University of Reading, Whiteknights, Reading, Berkshire, RG6 6AS, UK*

[3]*Department of Environment & Agriculture, Centre for Crop and Disease Management, Curtin University, Bentley 6102, Perth, Australia*

[*]**Author for correspondence - (ryan.sharp@rothamsted.ac.uk)**

Appendix S5. Roguing with replacement

It is common for smallholders to replace the plants that they remove through roguing, made possible by the plant's ability to be planted and harvested at various times in the year. We therefore modify the planting rate of the model, σ, to $\left(\sigma + \rho\big(I_e(x,t) + I_i(x,t)\big)\right)$ in the model with the endemic strain and $\left(\sigma + \rho I_i(x,t)\right)$ in the model with just the invader in equation 7 of the main text. To calculate the wave speed of the invader when the endemic strain is present, we replace the planting rate in equation 7 of Appendix S1 with $(\sigma + \rho \bar{I}_e)$. Performing the linearisation on the model with the invasive strain only reduced to the original model. Roguing with replacement is therefore not expected to have an effect on speed in this case. We checked this result with the simulations, and the wave speeds agree with each other.

Despite this model leading to much higher levels of disease. The effect on the invaders rate of spread is similar to the original model. We still observe an increase in the rate of spread of the invasive strain with increased control when the endemic strain is present.

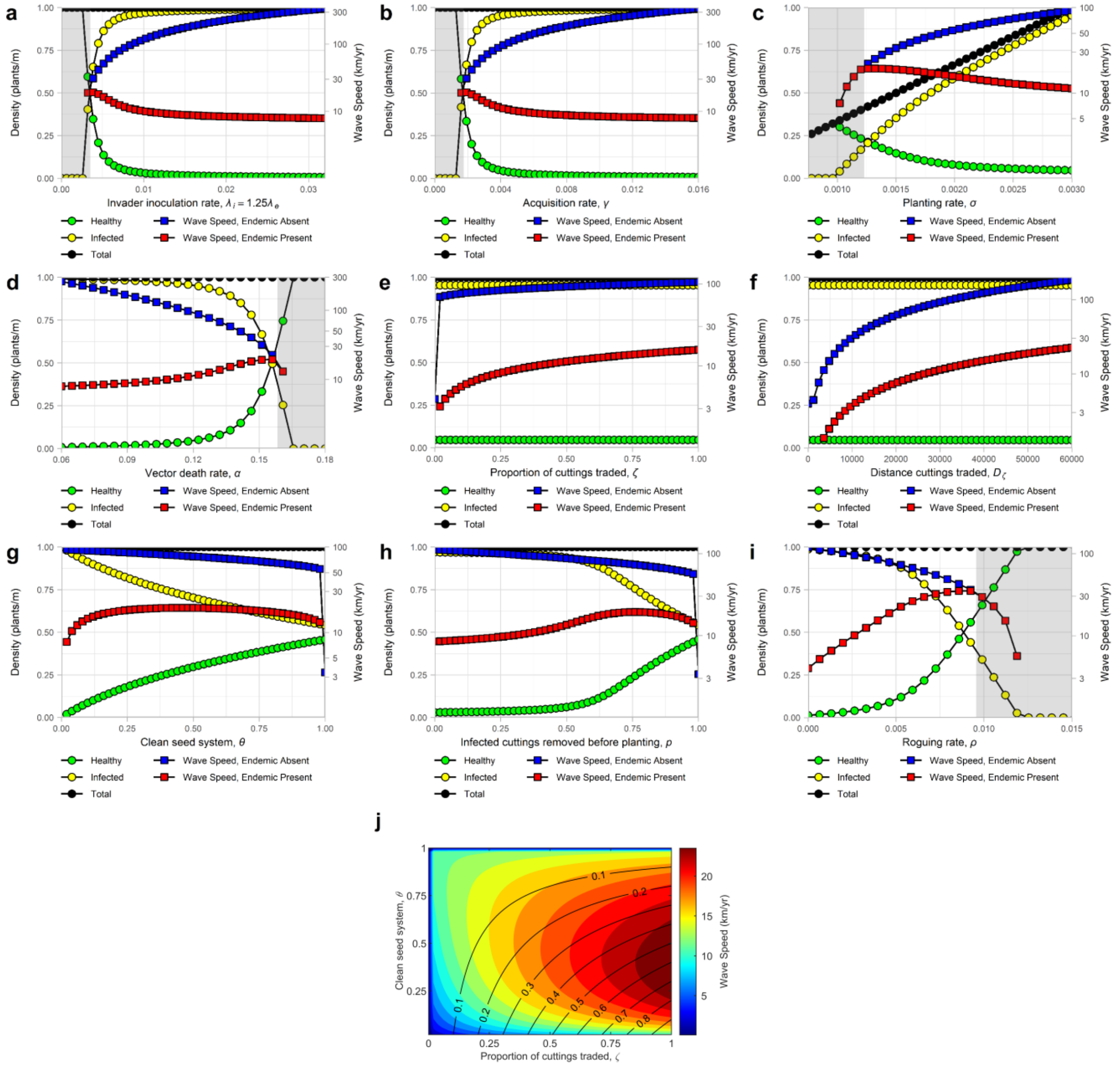

Figure 1 – One-way sensitivity analyses investigating the effect of immediately replacing rogued plants with cuttings while making changes to (a) the inoculation rate, λ and (b) the acquisition rate, γ, to model the planting of resistant cultivars; as well as (c) the planting rate, σ, to model crop abandonment; (d) the vector death rate, α; (e) the proportion of cuttings sourced through trade, ζ; (f) the standard deviation of the trade dispersal kernel, D_ζ; (g) the proportion of cuttings sourced through a clean seed system, θ; (h) the proportion of infected cuttings removed before planting, p; and, (i) the roguing rate, ρ; on: healthy ($H_{t=\infty}$, green), infected ($I_{i,t=\infty}$, yellow) and total (black) post-invasion host densities; and, speed of spread (log scale) of the invading pathogen strain when invading a region with the endemic strain present (red) and absent (blue). Figure (j) plots a two-way sensitivity analysis investigating the effect on invasion speed in the multi-strain model from changes in the proportion of cuttings sourced through either trade or a clean seed system. Black contours indicate the actual proportion of cuttings sourced through trade. The white points indicate missing data.
