# Supplementary material for: The effect of competition on the control of invading plant pathogens
Source: J Appl Ecol. 2020 Apr 17;57(7):1403–12. doi: 10.1111/1365-2664.13618 (PMC7386929; doi:10.1111/1365-2664.13618)
Supplement: Supplementary file 6 — Appendix S6 [file JPE-57-1403-s006.pdf]

The effect of competition on the control of invading plant pathogens

Ryan T. Sharp[1, *], Michael W. Shaw[2] & Frank van den Bosch[3]

[1]*Department of Sustainable Agriculture Sciences, Rothamsted Research, Harpenden, Hertfordshire, AL5 2JQ, UK*

[2]*School of Agriculture, Policy and Development, University of Reading, Whiteknights, Reading, Berkshire, RG6 6AS, UK*

[3]*Department of Environment & Agriculture, Centre for Crop and Disease Management, Curtin University, Bentley 6102, Perth, Australia*

[*]**Author for correspondence - (ryan.sharp@rothamsted.ac.uk)**

Appendix S6. Virus persistence & cassava brown streak disease

Cassava mosaic virus is a persistent virus, remaining in its vector for at least 9 days and sometimes for its entire lifetime (Dubern, 1994). Preliminary results, however, suggest that cassava brown streak virus (CBSV) is retained by the vector for only 24 hours (Maruthi, unpublished data; cited in: Legg *et al.*, 2011).

The CBSV pandemic began much more recently than the CMV pandemic. Data is therefore lacking to fully parameterise the model for this pathogen. A CBSV outbreak is modelled by using the default parameter set and introducing a virus loss rate, τ:

$$\begin{aligned}\frac{\partial Z_e(x,t)}{\partial t} &= -(\alpha+\tau)Z_e(x,t) + \gamma I_e(x,t)Y(x,t) + \delta_e(x,t),\\ \frac{\partial Z_i(x,t)}{\partial t} &= -(\alpha+\tau)Z_i(x,t) + \gamma I_i(x,t)Y(x,t) + \delta_i(x,t).\end{aligned} \quad 1$$

A much lower virus loss rate than is typical for cassava brown streak disease is used (virus loss after 10 days as opposed to 1) as higher rates quickly render the default parameter set unsuitable for the pathogen, implying that a better parameterisation of CBSV would be one less severe than that of CMV.

Due to the higher virus loss of CBSV, measures that attempt to reduce the spread of the virus via natural or artificial barriers such as borders where no host is grown will be more effective for CBSV than CMV. This can be observed in the results by comparing the effect of reducing planting rate when there is no virus loss in a vectors lifetime (figure 2 in Appendix S3) with that in figure 1.

CBSV appears to be limited by altitude/climate. The model does not factor this in and therefore should be taken as behaviour expected in regions that are suitable for the pathogen. Such dynamics could be accounted for in the simulations however if the parameters of the model were spatially heterogeneous.

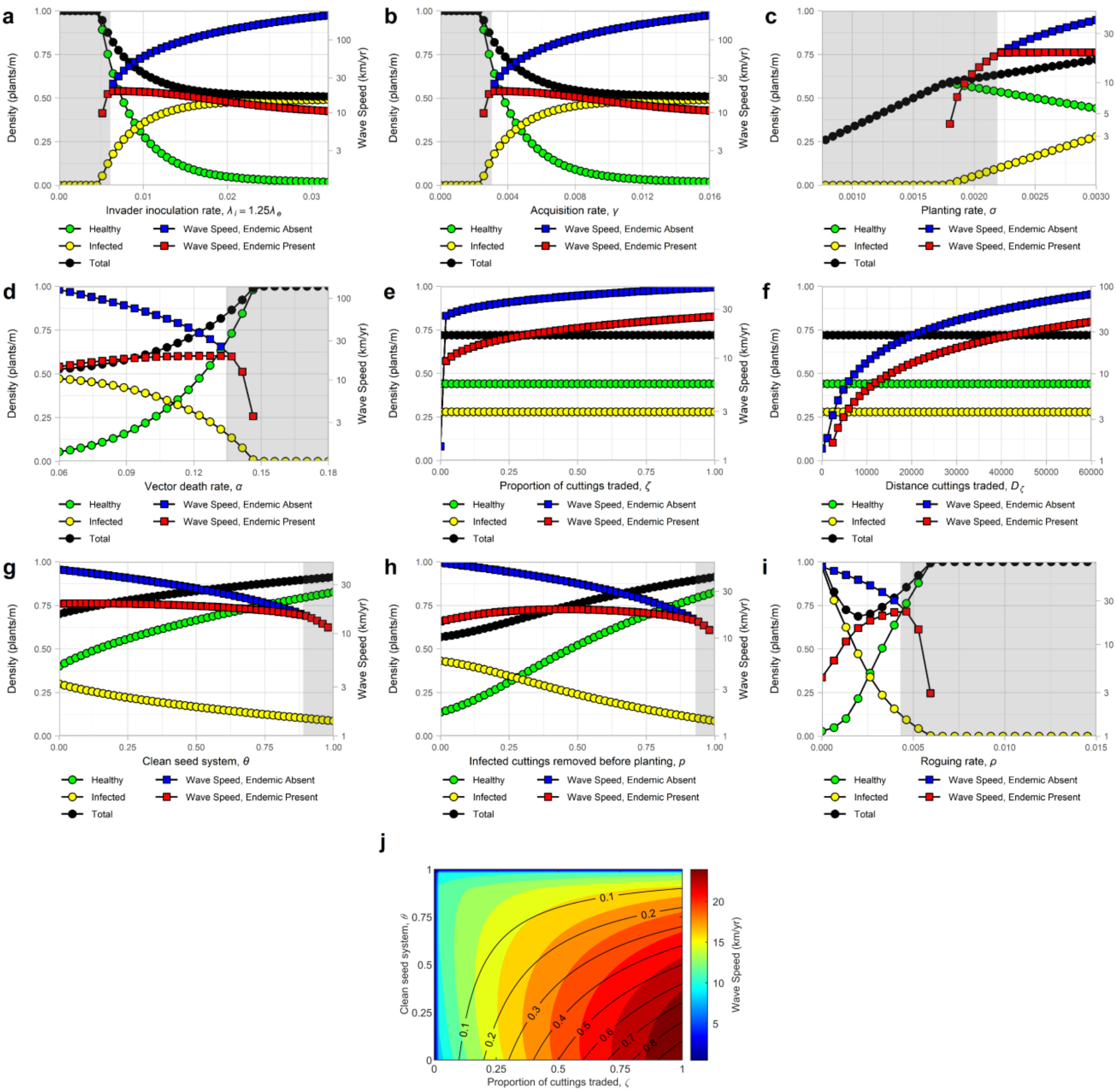

Figure 1 – One-way sensitivity analyses investigating the effect of incorporating virus loss (0.1 d^{-1}) from the vector while making changes to (a) the inoculation rate, λ and (b) the acquisition rate, γ, to model the planting of resistant cultivars; as well as (c) the planting rate, σ, to model crop abandonment; (d) the vector death rate, α; (e) the proportion of cuttings sourced through trade, ζ; (f) the standard deviation of the trade dispersal kernel, D_ζ; (g) the proportion of cuttings sourced through a clean seed system, θ; (h) the proportion of infected cuttings removed before planting, p; and, (i) the roguing rate, ρ; on: healthy, infected and total post-invasion host densities; and, speed of spread (log scale) of the invading pathogen strain when invading a region with the endemic strain present and absent. Figure (j) plots a two-way sensitivity analysis investigating the effect on invasion speed in the multi-strain model from changes in the proportion of cuttings sourced through either trade or a clean seed system. Black contours indicate the actual proportion of cuttings sourced through trade.

References

Dubern, J. (1994). Transmission of African cassava mosaic geminivirus by the whitefly (*Bemisia tabaci*). *Tropical Science, 34*(1)**,** 82-91.

Legg, J. P., Jeremiah, S. C., Obiero, H. M., Maruthi, M. N., Ndyetabula, I., Okao-Okuja, G., Bouwmeester, H., Bigirimana, S., Tata-Hangy, W., Gashaka, G., Mkamilo, G., Alicai, T. & Kumar, P. L. (2011). Comparing the regional epidemiology of the cassava mosaic and cassava brown streak virus pandemics in Africa. *Virus Research, 159*(2)**,** 161-170. https://doi.org/10.1016/j.virusres.2011.04.018.
