# Supplementary material for: The effect of competition on the control of invading plant pathogens
Source: J Appl Ecol. 2020 Apr 17;57(7):1403–12. doi: 10.1111/1365-2664.13618 (PMC7386929; doi:10.1111/1365-2664.13618)
Supplement: Supplementary file 7 — Appendix S7 [file JPE-57-1403-s007.pdf]

The effect of competition on the control of invading plant pathogens

Ryan T. Sharp[1,*], Michael W. Shaw[2] & Frank van den Bosch[3]

[1]*Department of Sustainable Agriculture Sciences, Rothamsted Research, Harpenden, Hertfordshire, AL5 2JQ, UK*

[2]*School of Agriculture, Policy and Development, University of Reading, Whiteknights, Reading, Berkshire, RG6 6AS, UK*

[3]*Department of Environment & Agriculture, Centre for Crop and Disease Management, Curtin University, Bentley 6102, Perth, Australia*

[*]**Author for correspondence - (ryan.sharp@rothamsted.ac.uk)**

Appendix S7. Frequency-dependent disease transmission

In this paper the simplifying assumption was made that disease transmission is density-dependent. However EACMV-UG is transmitted by a vector. In such cases disease transmission tends to be, or is better modelled using frequency-dependence. The existing model is modified to include frequency-dependent disease transmission:

$$\begin{aligned}
\frac{\partial H(x,t)}{\partial t} &= \varphi_H(x,t) - \omega H(x,t) - \frac{\lambda_e Z_e(x,t)H(x,t)}{H(x,t)+I_e(x,t)+I_i(x,t)} - \frac{\lambda_i Z_i(x,t)H(x,t)}{H(x,t)+I_e(x,t)+I_i(x,t)},\\
\frac{\partial I_e(x,t)}{\partial t} &= \varphi_{I_e}(x,t) + \frac{\lambda_e Z_e(x,t)H(x,t)}{H(x,t)+I_e(x,t)+I_i(x,t)} - (\omega+\rho)I_e(x,t),\\
\frac{\partial I_i(x,t)}{\partial t} &= \varphi_{I_i}(x,t) + \frac{\lambda_i Z_i(x,t)H(x,t)}{H(x,t)+I_e(x,t)+I_i(x,t)} - (\omega+\rho)I_i(x,t),\\
\frac{\partial Z_e(x,t)}{\partial t} &= -\alpha Z_e(x,t) + \frac{\gamma I_e(x,t)Y(x,t)}{H(x,t)+I_e(x,t)+I_i(x,t)} + \delta_e(x,t),\\
\frac{\partial Z_i(x,t)}{\partial t} &= -\alpha Z_i(x,t) + \frac{\gamma I_i(x,t)Y(x,t)}{H(x,t)+I_e(x,t)+I_i(x,t)} + \delta_i(x,t).
\end{aligned} \tag{1}$$

To calculate the wave speed the coefficient matrix, A, of Appendix S1 becomes:

$$A = \begin{pmatrix} \dfrac{\sigma(1-\theta)(1-p)}{\bar{H}+(1-p)\bar{I}_e}\left[(1-\zeta)+\zeta\hat{f}_\zeta(a)\right]-(\omega+\rho) & \dfrac{\lambda_i\bar{H}}{\bar{H}+\bar{I}_e} \\ \dfrac{\gamma_i(P-\bar{Z}_e)}{\bar{H}+\bar{I}_e} & -\alpha+m\left[\hat{f}_i(a)-1\right] \end{pmatrix}. \tag{2}$$

Results were found to be largely consistent with the results from the density-dependent model. Two issues did arise however. The first is the high levels of infection observed in the model. Parameters of the model such as the total vector density for instance, was chosen to represent the levels observed during the EACMV-UG epidemic in Uganda. This epidemic was particularly severe, and therefore the high levels of infection predicted by the frequency-dependent model could be just as viable a prediction as that predicted by the density-dependent model. The other issue is the peculiar dynamics observed when making changes

to the planting rate, σ. Contrary to all other results presented here, as control is increased, by decreasing the planting rate, the invasion speed of the invader decreases in the presence of an endemic strain and increases when there is no endemic strain present. This can at least in part be explained by the lack of healthy host, as the force of infection is so strong, even the weaker endemic strain manages to infect the entire population before the invasive strain has been introduced. Increasing control can be insufficient for the endemic strain to relinquish any of the healthy host. This therefore means that the invader receives no benefit from control and the net effect is therefore to suppress the growth of the invader. The reason for the increase in the speed of spread of the invader in the absence of an endemic strain is due to the assumption of a constant vector population. As opposed to the vectors in density-dependent models that when faced with a food shortage, such as that caused when planting rate is decreased and total host density is reduced, essentially reduce the amount of food that they consume, for a vector population to remain constant under the assumptions of frequency-dependence, the vectors will either need to increase in density on the available host remaining, if any, or seek additional hosts elsewhere. In the model however the vector is restricted to the former case as both the migration rate and mean dispersal distance are both fixed parameters of the model and no upper limit on the maximum vector density per unit plant area has been set. This increased density per unit plant area therefore greatly increases the transmissibility of the disease by increasing the inoculation and acquisition rates of the model. It is likely then that relaxing the assumption of constant vector densities would return levels of disease to levels similar to those observed in the density-dependent model as vector populations would be lower in systems with increased disease as, due to the roguing of infected hosts, host levels would be lower.

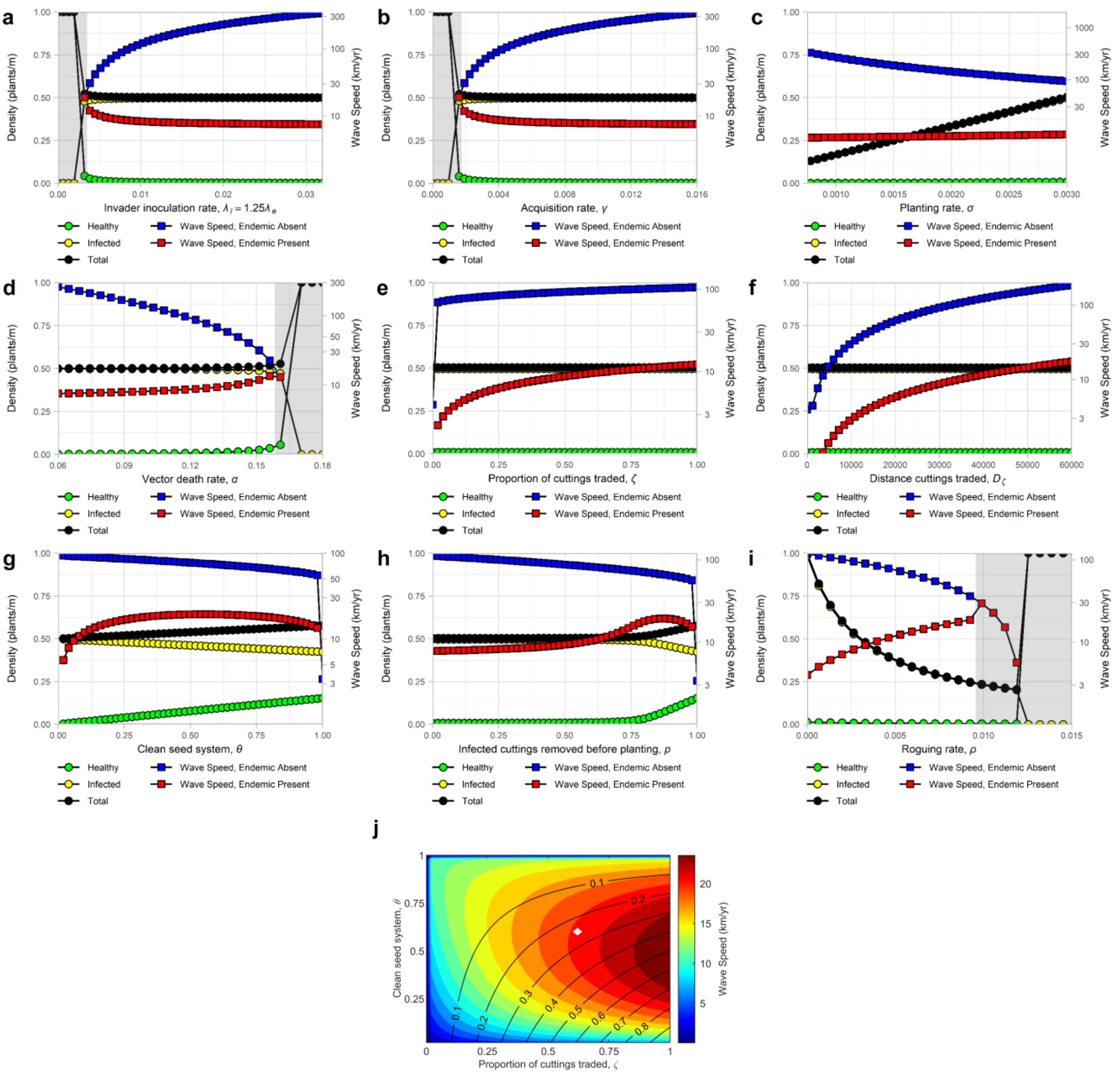

Figure 1 – One-way sensitivity analyses investigating the effect of frequency-dependent disease transmission while making changes to (a) the inoculation rate, λ and (b) the acquisition rate, γ, to model the planting of resistant cultivars; as well as (c) the planting rate, σ, to model crop abandonment; (d) the vector death rate, α; (e) the proportion of cuttings sourced through trade, ζ; (f) the standard deviation of the trade dispersal kernel, D_ζ; (g) the proportion of cuttings sourced through a clean seed system, θ; (h) the proportion of infected cuttings removed before planting, p; and, (i) the roguing rate, ρ; on: healthy, infected and total post-invasion host densities; and, speed of spread (log scale) of the invading pathogen strain when invading a region with the endemic strain present and absent. Figure (j) plots a two-way sensitivity analysis investigating the effect on invasion speed in the multi-strain model from changes in the proportion of cuttings sourced through either trade or a clean seed system. Black contours indicate the actual proportion of cuttings sourced through trade. The white point indicates missing data.
