# Supplementary material for: The effect of competition on the control of invading plant pathogens
Source: J Appl Ecol. 2020 Apr 17;57(7):1403–12. doi: 10.1111/1365-2664.13618 (PMC7386929; doi:10.1111/1365-2664.13618)
Supplement: Supplementary file 8 — Appendix S8 [file JPE-57-1403-s008.pdf]

The effect of competition on the control of invading plant pathogens

Ryan T. Sharp[1, *], Michael W. Shaw[2] & Frank van den Bosch[3]

[1]*Department of Sustainable Agriculture Sciences, Rothamsted Research, Harpenden, Hertfordshire, AL5 2JQ, UK*

[2]*School of Agriculture, Policy and Development, University of Reading, Whiteknights, Reading, Berkshire, RG6 6AS, UK*

[3]*Department of Environment & Agriculture, Centre for Crop and Disease Management, Curtin University, Bentley 6102, Perth, Australia*

[*]**Author for correspondence - (ryan.sharp@rothamsted.ac.uk)**

Appendix S8. Increased detection of the invasive strain

It is possible, due to the invasive strain's fitness advantage over the endemic strain, that this fitness advantage could also lead to the strain being more easily detected. Here we investigate the effect of increased roguing on the invasive strain, with the roguing rate of the invasive strain set to be roughly 10% higher than the endemic strain figure 1. We also investigate the case in which the roguing rate of the invasive strain is kept at the default value, but control of the endemic strain is relaxed figure 2. We observe lower rates of spread of the invader in both cases, in the former case this is due to increased control on the invasive strain, in the latter it is due to the increased effect of competition from the endemic strain. The rate of spread of the invasive strain still increases with increased control. With low levels of control, we begin to reach a point where endemic strain can coexist with the invasive strain. As control is decreased further the invasive strain is no longer able to invade.

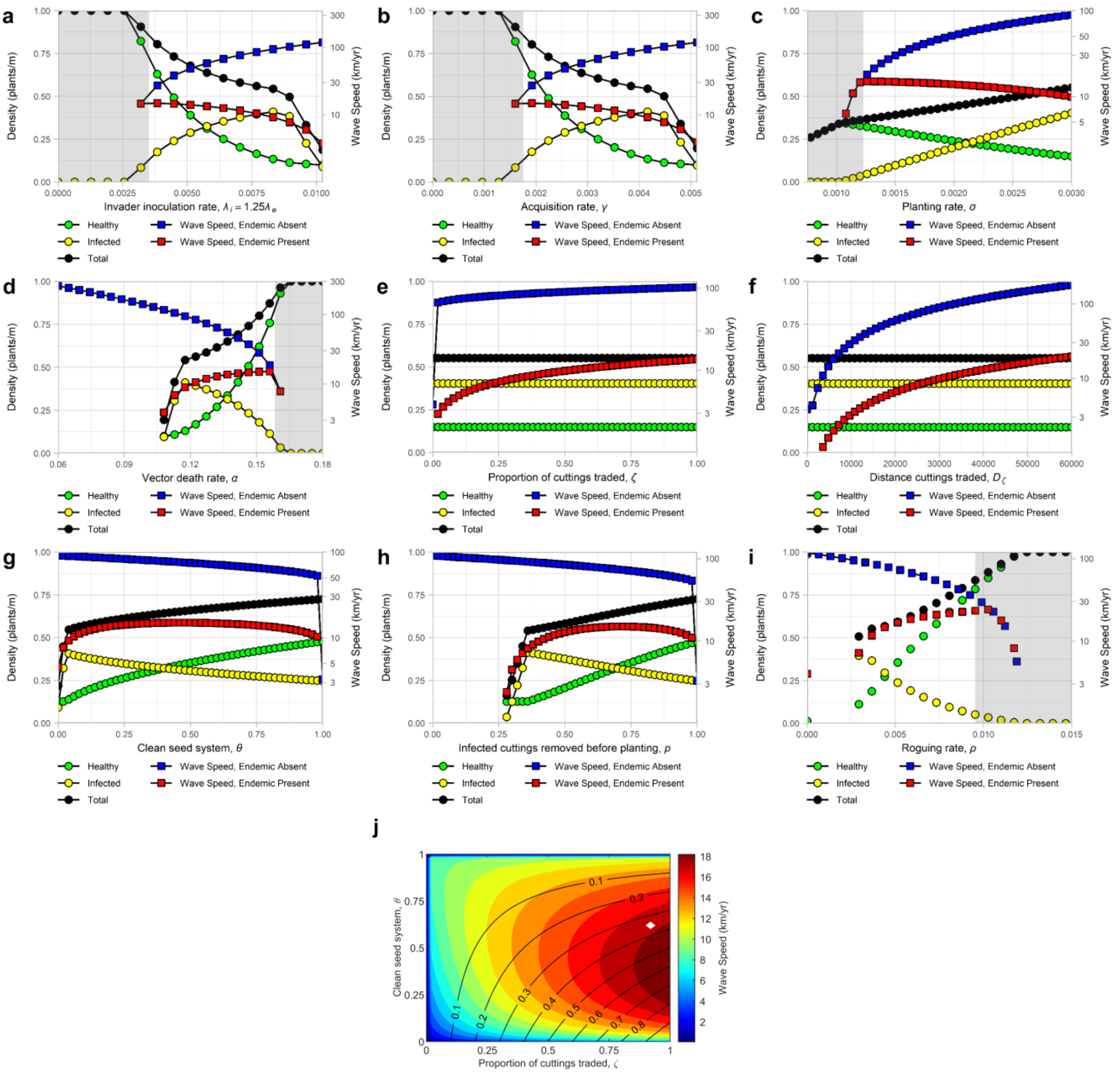

Figure 1 – One-way sensitivity analyses investigating the effect of an increased roguing rate of the invasive strain due to the strain being more easily detected while making changes to (a) the inoculation rate, λ and (b) the acquisition rate, γ, to model the planting of resistant cultivars; as well as (c) the planting rate, σ, to model crop abandonment; (d) the vector death rate, α; (e) the proportion of cuttings sourced through trade, ζ; (f) the standard deviation of the trade dispersal kernel, D_ζ; (g) the proportion of cuttings sourced through a clean seed system, θ; (h) the proportion of infected cuttings removed before planting, p; and, (i) the roguing rate, ρ; on: healthy, infected and total post-invasion host densities; and, speed of spread (log scale) of the invading pathogen strain when invading a region with the endemic strain present and absent. Figure (j) plots a two-way sensitivity analysis investigating the effect on invasion speed in the multi-strain model from changes in the proportion of cuttings sourced through either trade or a clean seed system. Black contours indicate the actual proportion of cuttings sourced through trade. The white point indicates missing data.

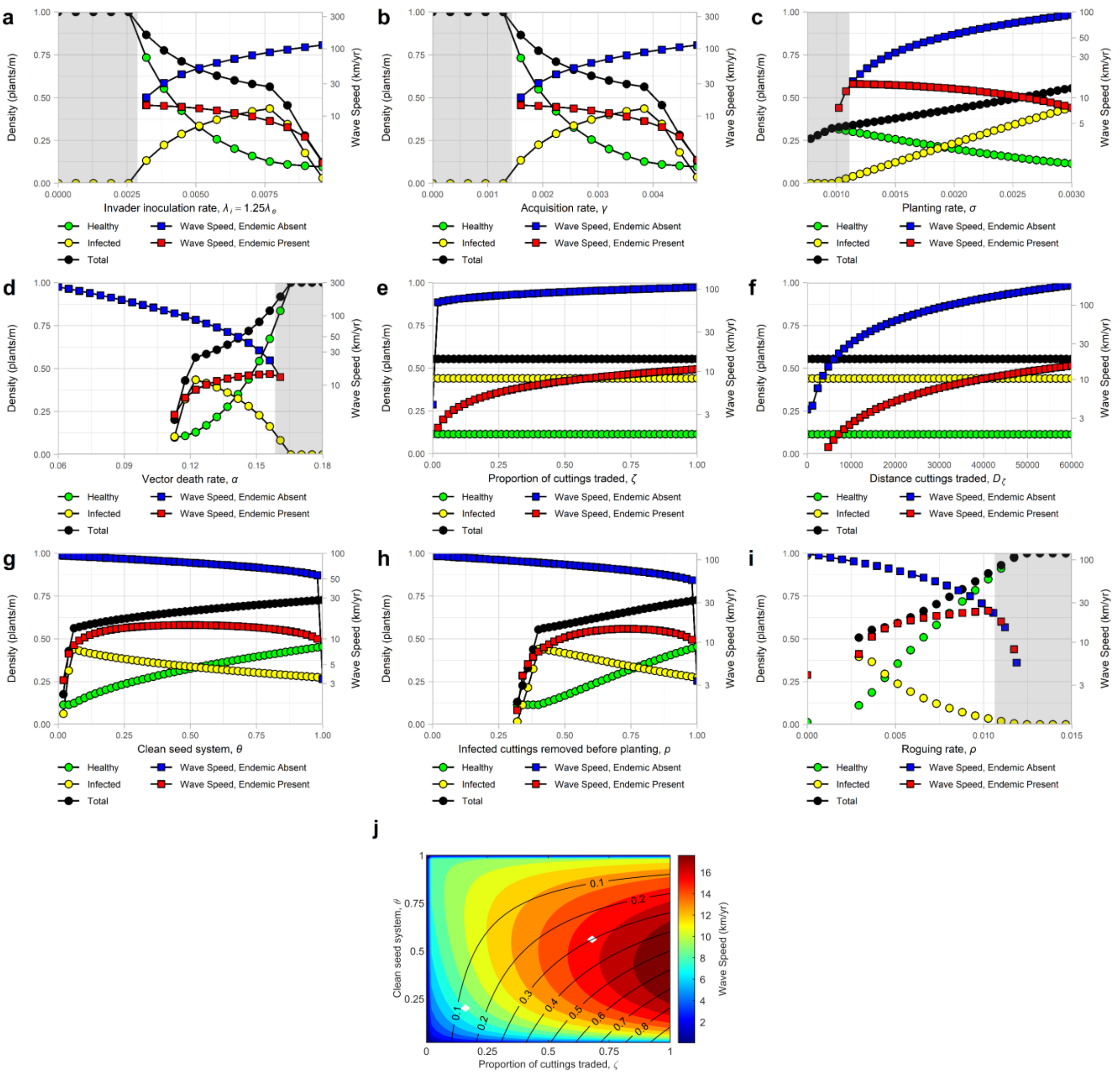

Figure 2 – One-way sensitivity analyses investigating the effect of a decreased roguing rate of the endemic strain due to the invasive strain being more easily detected while making changes to (a) the inoculation rate, λ and (b) the acquisition rate, γ, to model the planting of resistant cultivars; as well as (c) the planting rate, σ, to model crop abandonment; (d) the vector death rate, α; (e) the proportion of cuttings sourced through trade, ζ; (f) the standard deviation of the trade dispersal kernel, D_ζ; (g) the proportion of cuttings sourced through a clean seed system, θ; (h) the proportion of infected cuttings removed before planting, p; and, (i) the roguing rate, ρ; on: healthy, infected and total post-invasion host densities; and, speed of spread (log scale) of the invading pathogen strain when invading a region with the endemic strain present and absent. Figure (j) plots a two-way sensitivity analysis investigating the effect on invasion speed in the multi-strain model from changes in the proportion of cuttings sourced through either trade or a clean seed system. Black contours indicate the actual proportion of cuttings sourced through trade. The white point indicates missing data.
