# Supplementary material for: The effect of competition on the control of invading plant pathogens
Source: J Appl Ecol. 2020 Apr 17;57(7):1403–12. doi: 10.1111/1365-2664.13618 (PMC7386929; doi:10.1111/1365-2664.13618)
Supplement: Supplementary file 9 — Appendix S9 [file JPE-57-1403-s009.pdf]

The effect of competition on the control of invading plant pathogens

Ryan T. Sharp[1, *], Michael W. Shaw[2] & Frank van den Bosch[3]

[1]*Department of Sustainable Agriculture Sciences, Rothamsted Research, Harpenden, Hertfordshire, AL5 2JQ, UK*

[2]*School of Agriculture, Policy and Development, University of Reading, Whiteknights, Reading, Berkshire, RG6 6AS, UK*

[3]*Department of Environment & Agriculture, Centre for Crop and Disease Management, Curtin University, Bentley 6102, Perth, Australia*

[*]**Author for correspondence - (ryan.sharp@rothamsted.ac.uk)**

Appendix S9. Effect of the relative difference between strains

We have so far distinguished the invasive strain from the endemic strain by assuming that the invasive inoculation rate is 1.25 times greater than the endemic. Here we relax that assumption and model two new cases. One where the invasive strain's inoculation rate differs by 1.1 times the endemic figure 1, and another where it differs by 1.5 times figure 2. We see that in the former case, we don't always observe a decrease to the invasive strains speed in the grey regions of the plot. This is because, due to the small difference between strains, both strains die out at roughly the same time. The grey region is also greater when there is greater difference between strains, as the invasive strain can then withstand greater levels of control. We still observe the increase in the invaders speed with increasing control when the endemic strain is present in both cases tested.

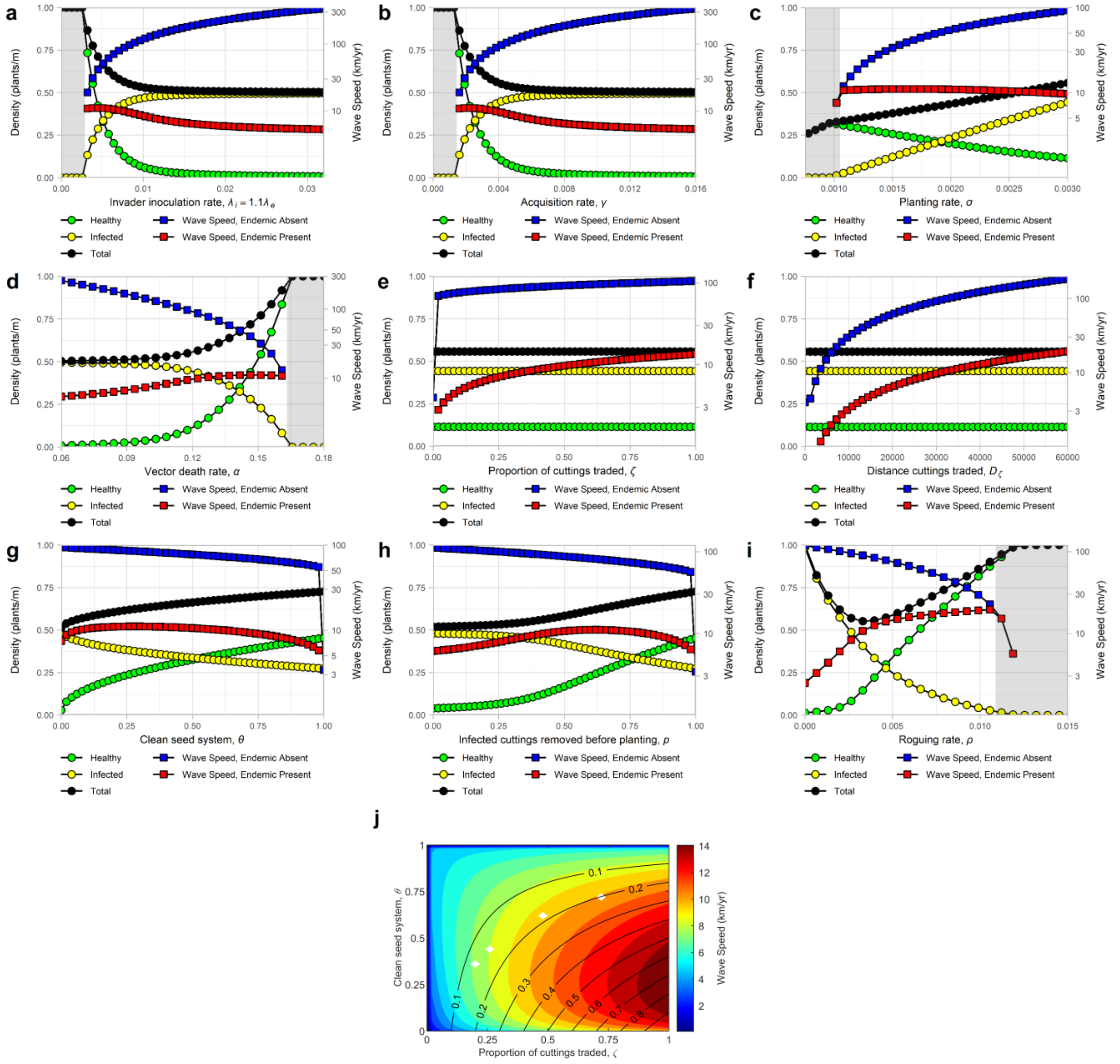

Figure 1 – One-way sensitivity analyses investigating the effect of reducing the advantage of the invasive strain to 1.1 times the endemic's inoculation rate while making changes to (a) the inoculation rate, λ and (b) the acquisition rate, γ, to model the planting of resistant cultivars; as well as (c) the planting rate, σ, to model crop abandonment; (d) the vector death rate, α; (e) the proportion of cuttings sourced through trade, ζ; (f) the standard deviation of the trade dispersal kernel, D_ζ; (g) the proportion of cuttings sourced through a clean seed system, θ; (h) the proportion of infected cuttings removed before planting, p; and, (i) the roguing rate, ρ; on: healthy, infected and total post-invasion host densities; and, speed of spread (log scale) of the invading pathogen strain when invading a region with the endemic strain present and absent. Figure (j) plots a two-way sensitivity analysis investigating the effect on invasion speed in the multi-strain model from changes in the proportion of cuttings sourced through either trade or a clean seed system. Black contours indicate the actual proportion of cuttings sourced through trade. The white point indicates missing data.

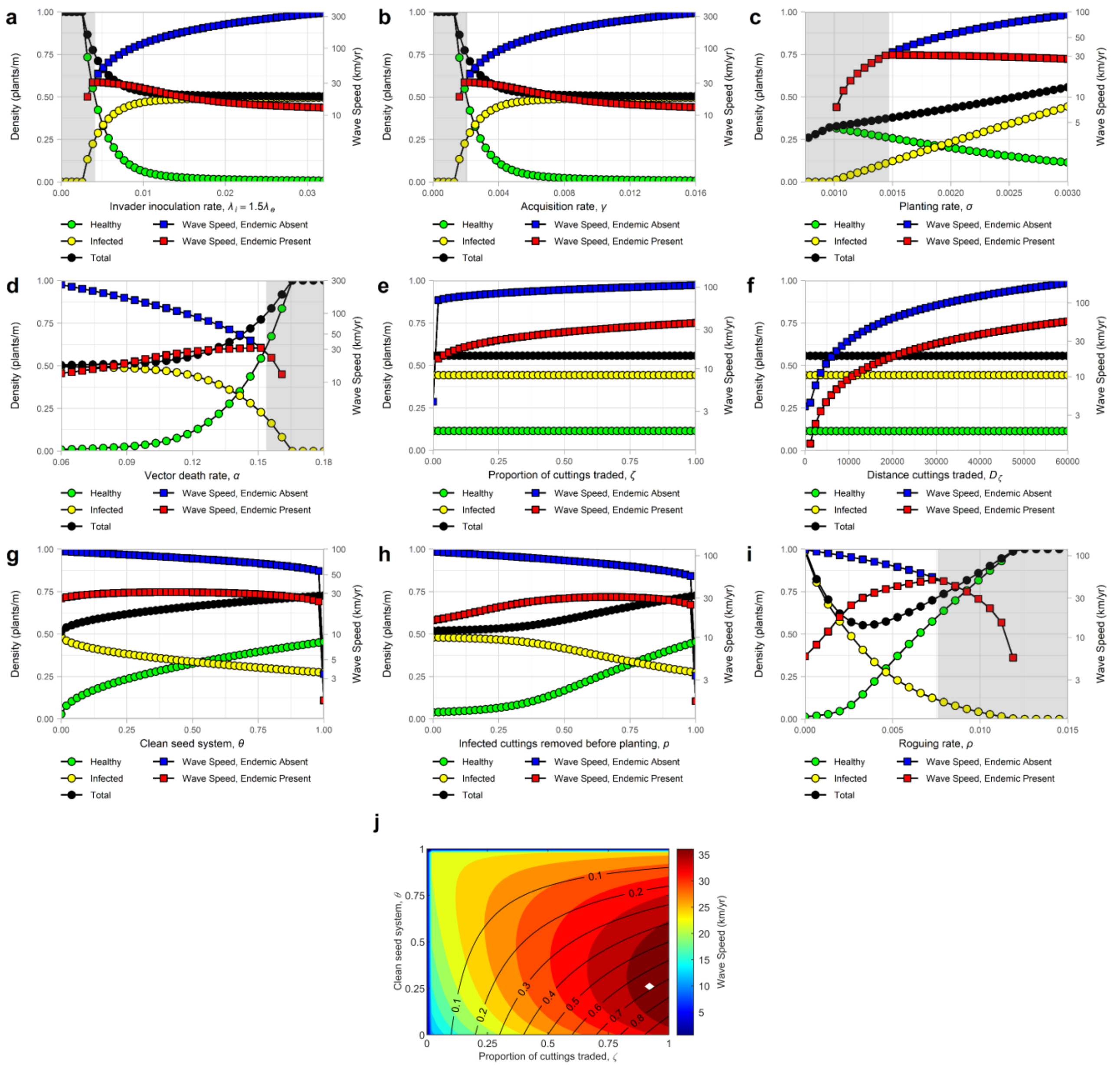

Figure 2 – One-way sensitivity analyses investigating the effect of increasing the advantage of the invasive strain to 1.5 times the endemic's inoculation rate while making changes to (a) the inoculation rate, λ and (b) the acquisition rate, γ, to model the planting of resistant cultivars; as well as (c) the planting rate, σ, to model crop abandonment; (d) the vector death rate, α; (e) the proportion of cuttings sourced through trade, ζ; (f) the standard deviation of the trade dispersal kernel, D_ζ; (g) the proportion of cuttings sourced through a clean seed system, θ; (h) the proportion of infected cuttings removed before planting, p; and, (i) the roguing rate, ρ; on: healthy, infected and total post-invasion host densities; and, speed of spread (log scale) of the invading pathogen strain when invading a region with the endemic strain present and absent. Figure (j) plots a two-way sensitivity analysis investigating the effect on invasion speed in the multi-strain model from changes in the proportion of cuttings sourced through either trade or a clean seed system. Black contours indicate the actual proportion of cuttings sourced through trade. The white point indicates missing data.
